# Biological Potential and Physicochemical Properties of Ionic Liquids Bioinspired by Carboxylic Acids: A Review

**DOI:** 10.3390/ph19040570

**Published:** 2026-04-02

**Authors:** Carmen Terán, Marta María Mato, Pedro Besada

**Affiliations:** 1 Departamento de Química Orgánica, Universidade de Vigo, 36310 Vigo, Spain; pbes@uvigo.gal; 2 Instituto de Investigación Sanitaria Galicia Sur, Hospital Álvaro Cunqueiro, 36213 Vigo, Spain; 3 Departamento de Física Aplicada, Universidade de Vigo, 36310 Vigo, Spain

**Keywords:** carboxylic acids, bioinspired ILs, synthesis, biological potential, toxicity, physicochemical properties

## Abstract

Ionic liquids (ILs) derived from bioactive compounds have emerged as a versatile and highly tunable platform for designing novel functional materials with biomedical applications. Many of these systems incorporate naturally occurring carboxylate anions of relevance to medicinal chemistry, biotechnology, and biomedicine, which has intensified interest in this family of bioinspired ILs. This review focuses on ILs derived from carboxylic acids of natural origin, including fatty acids, phenolic acids, and hydroxy acids, and highlights recent advances in their design, bioactivity, and physicochemical characterization, with particular emphasis on systems based on biocompatible components. Additionally, it addresses synthetic strategies, toxicological aspects, and biological potential. Key physicochemical properties discussed include thermal stability, glass transition temperatures, melting and crystallization points, viscosity, density, solubility, refractive index, polarity, and amphiphilic behavior.

## 1. Introduction

Ionic liquids (ILs) are a particular class of molten salts which are liquids at relatively low temperature. There is a consensus among the scientific community in defining ILs as chemical compounds composed exclusively of ions, which are liquid at temperatures not exceeding 100 °C [1]. Some of them are called room-temperature ILs (RTILs) due to their liquid state close to this temperature [2,3].

Researcher’s interest in ILs arose in the second half of the 1990s, as they were considered not only a very good alternative to volatile organic solvents in several chemical processes, but also as catalysts [1,3,4,5,6]. One of the main goodness of the ionic liquids is their low vapor pressure, which has been associated with reduced environmental pollution and toxicity, and hence their consideration as green solvents [4]. This was also supported by its non-flammability and thermal stability [7]. However, the global classification of ILs as green solvents is questioned, probably because of their chemical stability and water solubility, two factors that can be potential risks to the environment and living beings. In this regard, and given the broad concept of IL, the toxicological and ecotoxicological data currently available remain very scarce [8,9,10].

In ILs the cation is usually a bulky asymmetric organic cation, and the anion displays weak coordination properties resulting in a crystal lattice more destabilized than that of classical organic salts. The low symmetry in the cation along with the charge delocalization and its ability of interaction with the corresponding anion are key features in RTILs. The anion size also influences the melting point values, it is usually a polyatomic ion; as a rule, for a given charge, an increase in the anion magnitude leads to a decrease in salt melting point [11,12].

**Figure 1:**
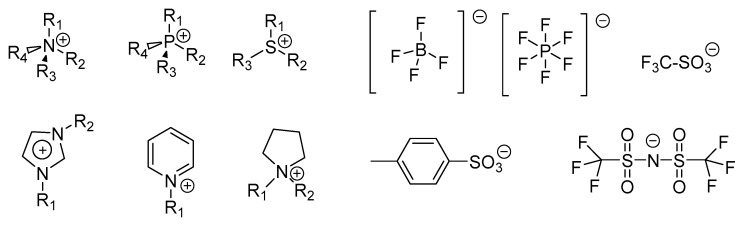
A selection of cations and anions commonly used in RTILs.

In the best known RTILs the cation is commonly a tetraalkylammonium, tetralkylphosphonium, triakylsulfonium or a heterocyclic derivative, such as 1,3-dialkyl-imidazolium, N-alkylpyridinium or N, N-dialkylpyrrolidinium, among others. These are combined with an inorganic or organic anion, for instance, tetrafluroborate, hexafluorophosphate, trifluoromethanesulfonate, *p*-toluensulfonate or bis(trifluoromethanesulfonyl)imide [12,13] (Figure 1). The possibility of combining different cations and anions allows obtaining a huge number of ILs. In addition, through an appropriate modification of the cation and/or the anion, it is possible to modulate certain chemical and physics properties, such as polarity, hydrophobicity, liquid range, heat capacity, viscosity, solvent miscibility or even recyclability, which can be determinants of the efficiency of a reaction solvent. All these aspects have contributed to qualifying ILs as designer solvents [4].

Beyond their recognized qualities as solvents, the customizable nature of ILs has facilitated the development of a plethora of novel applications in many areas and topics, not only in chemistry [14,15,16], but also in physics [17,18], biotechnology [19,20], engineering [21] or biomedicine [22,23,24], among others.

The first studies linking ionic liquids and biomedicine emerged at the beginning of the 2000s, emphasizing their usefulness in drug synthesis and biocatalysis [25,26,27,28,29]. In these two fields, ILs are mainly described as solvents and catalysts, where they can potentially affect the reaction regioselectivity, often improving the yield, shortening the reaction time and simplifying the reaction workup. Despite these recognized advantages, the pharmaceutical industry has questioned their use as solvents, particularly in relation to purity, toxicity and regulatory criteria [30].

A few years later, the use of ILs as active pharmaceutical ingredients (APIs) was proposed by R. Rogers et al. as an alternative approach to crystalline salts [31,32] to avoid the possibility of polymorphs or solvates formation. This is because the kind of solid structure determines important properties, such as solubility, bioavailability or even pharmacokinetics, which may directly affect drug efficacy. Additional advantages of the API ionic liquids (API-ILs) approach lie in the possibility of combining two biologically active species (cation and anion) to achieve synergistic or complementary effects; lidocainium docusate is a representative example [32]. Moreover, this approach could help neutralize certain side effects attributed to one of the ionic components [33]. Research on the biolo-gical activity of ILs has grown significantly since these initial proposals [22,34,35,36], extending also to the drug delivery field [34,37]. Considering the current need for more effective and innovative therapeutic strategies, the development of ILs in medicinal chemistry will continue to be a highly attractive area of research in the coming years.

ILs are also described as good solvents for extracting bioactive compounds from plants [38]. Natural products constitute a valuable source of inspiration in drug discovery. The structural complexity and diversity of natural products, with a predominance of aliphatic rings over aromatic ones and a higher number of tetrahedral carbons and chiral centers than synthetic compounds [39], could explain their good ability to interact with a wide variety of biological targets. The biological activities reported for natural products are very diverse, including anticancer [40], antimicrobial [41], anti-inflammatory [42], antioxidant [43], and neuroprotective effects [44], among many others [45]. Consequently, they can be an excellent starting point for obtaining new APIs. However, some natural products display poor bioavailability, a pharmacokinetic property related to the solubility and membrane permeability of APIs, which affects absorption rate and limits their activity and clinical potential.

Effective absorption requires a delicate balance as the compound must dissolve adequately in aqueous biological fluids while maintaining sufficient lipophilicity to cross lipid-rich cellular membranes. Highly hydrophilic molecules often fail in this process, whereas excessively lipophilic compounds may show poor dissolution and an inadequate absorption profile [46].

Structural modifications are therefore often essential to improve the bioactive profile of promising natural products. These structural changes, frequently involving obtaining salts and/or semi-synthetic derivatives, including prodrugs, can provide a new chemical entity that retains the structural elements responsible for the biological activity in the original natural product [47].

The conversion of an API into a salt is a common way to improve solubility and dissolution rate, which could enhance its bioavailability. When the resulting salt is a liquid, issues related to polymorphism are also avoided. In this context, API-ILs offer a versatile platform to adjust these properties by combining the API with a counterion that modulates polarity and amphiphilic character. The rational selection of cations and anions enables the improvement of aqueous solubility without compromising membrane permeability, thereby optimizing parameters such as octanol/water partition coefficient (logP), an indicator of lipophilicity and membrane permeability, and polar surface area (PSA), a measure of polarity and hydrogen-bonding capacity, which would translate into more predictable pharmacokinetic profiles and enhanced therapeutic potential. Properties such as solubility, viscosity, surface tension, amphiphilic character, melting point, and thermal stability directly influence functionality and interactions with excipients and tissues. The relevance of each property depends on the intended therapeutic application [34].

In antimicrobial applications, amphiphilic properties such as critical micelle concentration (CMC) and surface tension (γ) are decisive, as they enable interaction with and the disruption of cell membranes, enhancing the biocidal action of the compound. Likewise, viscosity plays a key role in system handling and diffusion capacity, as appropriate viscosity promotes skin penetration in transdermal formulations and contributes to the stability of emulsions in food and cosmetic, ensuring both technological functionality and active ingredient efficacy [48,49,50].

Thermal stability is equally essential to ensure the integrity of API-ILs during storage and transportation, preventing degradation, loss of efficacy, and the formation of toxic byproducts [51]. Environmental factors such as humidity, temperature, and light can significantly influence these characteristics, making the application of analytical techniques such as differential scanning calorimetry (DSC), thermogravimetric analysis (TGA), and rheological measurements indispensable for comprehensive evaluation [52]. Furthermore, physicochemical parameters including density and refractive index are fundamental for characterization and quality control, while optical activity, present in ILs derived from chiral acids such as mandelic acid, may influence biological selectivity, although its impact could be in some cases less significant than amphiphilic properties [53,54].

All these properties define the technical viability of ionic liquids and allow the modulation of their therapeutic efficacy, toxicity, and pharmacokinetic behavior, consolidating their role as innovative tools in drug design and delivery systems [23,34].

Many natural products can be transformed into ILs by means of straightforward procedures, such as simple ion metathesis or acid-base reactions. In this context, J. H. Davis Jr. et al. described a remarkable example of a naturally occurring IL formed with a specific role when two belligerent ant species confront each other, by combining their toxins, the piperidine alkaloids (solenopsins) of *Solenopsis invicta* and the formic acid of *Nylanderia fulva* venom [55].

For some years now, the synthesis of ILs from bioactive compounds has emerged as an attractive strategy for researchers working in natural products, since it allows access to new APIs in a biosustainable way. Most ILs derived from natural products, commonly referred to as bioinspired or bio-based ILs, contain ions derived from choline, amino acids, carbohydrates, terpenes or carboxylic acids. Several reviews have analyzed the remarkable chemical and biological potential of bioinspired ILs [56,57,58,59,60]. The most recent ones have mainly focused on sugars [61,62,63], choline, amino acids and terpenes [58,60,62,64] as sources of ILs and to a lesser extent on carboxylic acids [58]. However, there is a considerable amount of literature available describing the various applications and properties of carboxylic acid-derived ILs. In this regard, it should be noted that naturally occurred carboxylic acids can be considered privileged bioactive molecules due to their therapeutic effects, while also serving as a valuable source of anions [65].

Consequently, this review discusses progress in bioactivity of ILs inspired by naturally occurring carboxylic acids over the past sixteen years (2010–March 2026), highlighting their potential as APIs, as well as analyzing their synthetic procedures and physicochemical properties. Deep eutectic solvent (DES) systems, typically composed of a hydrogen bond acceptor and a molecular hydrogen bond donor, and most used in extraction processes [66,67], are not the focus of this study.

## 2. Carboxylic Acid-Based ILs

Carboxylic acids, along with their esters and salts, are widely distributed in nature. Among naturally occurring carboxylic acids, aliphatic acids, commonly known as fatty acids, phenolic acids and related aliphatic and aromatic acids are very abundant, and many of them are interesting as anions for the design of bio-based ILs.

Quaternary ammonium salts displaying lipophilic alkyl groups are an important group of antiseptics and disinfectants. They are cationic surfactants with the ability to destroy bacteria and fungi while showing low toxicity toward homeothermic organisms. Many of these compounds are quaternary ammonium halides and through a simple anion exchange can be converted into aprotic ILs with specific physicochemical and antimicrobial properties.

It is well known that the two components of the IL, cation and anion, can play an important role in its toxicity [68]. In this context, it is worth mentioning that several natural carboxylic acids, specifically fatty acids, are biodegradable products of low toxicity and their alkaline salts, the soaps, are also effective surfactants [69]. Therefore, replacing the alkaline cation with a quaternary ammonium cation can provide liquid salts that retain this property. In addition, fatty acids are classified as generally recognized as safe (GRAS) by the U. S. Food and Drug Administration (FDA). All this has undoubtedly enhanced their interest as components of ILs.

ILs based on carboxylic acids can be classified into two main groups, aprotic and protic. In aprotic ILs, the cationic species arises from the incorporation of a group other than the proton into the basic part of the structure, which is often a nitrogen atom. In contrast, the formation of protic ILs (PILs) involves the transfer of a proton from an acid to a base. The PILs possess exchanging protons and therefore exhibit a certain degree of acidity that distinguishes them from aprotic ILs.

**Scheme 1:**
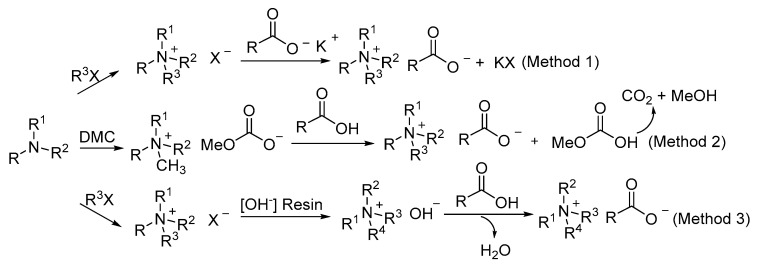
Synthesis methods of aprotic ammonium ILs containing carboxylate anions.

**Scheme 2:**
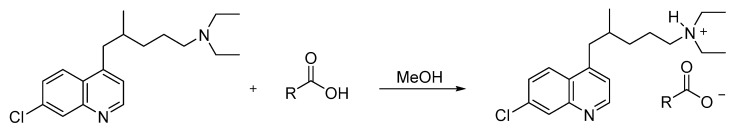
Synthesis of protic ammonium carboxylate ILs by direct acid-base neutralization.

Several strategies are commonly used to obtain alkyl ammonium ILs with carboxylate anions (Scheme 1 and Scheme 2).

To obtain aprotic ILs whose precursor cation is not commercially available, it is necessary to carry out the nitrogen quaternization, typically using an alkyl halide as the alkylating agent. Consequently, ammonium halides are the most common precursors of aprotic ILs [70]. Quaternization is followed by a metathesis reaction to introduce the anion. This second step can be performed using silver salts, Brønsted acids, or an alkali carboxylate (method 1). All three types of reagents present certain drawbacks: Silver salts are very expensive and their use is not feasible on a large scale; Brønsted acids generate acid halides as by-products, which are highly corrosive; and the use of alkali carboxylates, commonly employed to obtain aprotic ILs based on carboxylic acids from ammonium halides (method 1), produces inorganic salts as by-products, which are sometimes difficult to remove completely [71]. Washing water or extracting the IL with dichloromethane can give good results in the case of hydrophobic or hydrophilic ILs, respectively [71].

Likewise, it is worth noting that the use of alkyl halides as quaternization agents also presents limitations, since the precursor salt of the IL usually contains residual halide ions that could contaminate it. In this regard, dialkyl carbonates, such as dimethyl carbonate (DMC), are a good alternative as alkylating agents, since their salts, when subjected to the metathesis reaction with a Brønsted acid, generate the corresponding alkyl hydrogen carbonate as by-product [72], which breaks down into carbon dioxide and methanol (method 2).

Another alternative to obtain aprotic ILs from halogen-free intermediates consists of transforming ammonium halides into hydroxides using an [OH^−^] resin, and then neutra-lize the basic solution with the acidic counterpart [73]. This acid-basic neutralization provides the desired IL, avoiding the presence of contaminating inorganic salts (method 3).

Alternatively, PILs are obtained through direct acid-base neutralization. This reaction involves the combination of equimolar amounts of acid and base, typically requiring the gradual addition of acid to the basic species, usually an amine or an alkaloid [74] (Scheme 2). Furthermore, as the reaction is often strongly exothermic, temperature control is essential to prevent product degradation.

The most common impurities in PILs are the excess of acid or base. In this regard, some authors consider a PIL to be a pure salt when the proton transfer from the acid to the base reaches at least 99% [75]. Additionally, because PILs are generally more hygroscopic than aprotic ILs, water is another common contaminant. Therefore, various strategies have been developed to minimize the presence of moisture as well as excess acid or base, such as the simultaneous addition of pre-dried acid and base [76].

ILs derived from natural carboxylic acids combine organic cations and functionalized anions within their structure, promoting the formation of systems with low packing energy [1,11,12]. This structural feature translates into physical properties that differ from conventional salts, including high solubility, tunable viscosity, and thermal stability [23,24]. In general, these compounds may exist as stable liquids or amorphous phases with low or even negative glass transition temperatures (T_g_), which increases free energy and, consequently, enhance solubility and dissolution rate [31,32], key properties for improving the bioavailability of poorly soluble APIs. A representative example is cholinium propanoate [77], which exhibits an extremely low T_g_ (−74 °C), confirming the tendency of these systems to remain amorphous even at very low temperatures and to display high molecular mobility, thereby favoring solubility and dispersion. Amorphism eliminates the risk of polymorphism and allows adjustment of parameters such as colloidal behavior (particle size, zeta potential, surface tension, CMC, viscosity); however, it also entails lower physical stability and increased sensitivity to humidity and temperature, thus requiring strict storage conditions [8,10]. Conversely, crystalline phases offer greater stability and lower hygroscopicity but show limited solubility and a risk of polymorphism [33]. The choice between both states depends on balancing bioavailability and stability, making structural design a strategic tool for API optimization [22,34].

For the purposes of this study, ILs derived from naturally occurring carboxylic acids have been classified into three families, fatty acid-based ILs, phenolic acid-based ILs, and hydroxy acid-based ILs, considering both aprotic ILs and PILs.

### 2.1. Fatty Acid-Based ILs (FAILs)

The biological potential and biocompatibility of a variety of ILs containing alkanoate anions was studied by several researchers, in many cases exploring different aspects related to toxicity and environmental safety, as well as their potential as chemotherapeutic agents [74,77].

#### 2.1.1. Toxicological Aspects of FAILS

Currently, there is growing interest in ILs containing the cholinium ion, a functionalized cation regarded as environmentally friendly and low in toxicity, based on results obtained from studies in aquatic organisms and mammalian cell lines [8,68,78,79]. Accordingly, the FAILs studied by C. Silva-Pereira et al. [77] combine simple carboxylic acids containing between two (acetic acid) and ten (decanoic acid) carbon atoms, some of which exhibit branched structures, such as 2-methyl propanoic and 2,2-dimethylpropanoic acids, with the biocompatible cholinium cation. The target compounds were synthesized using an acid-base neutralization strategy, in which an aqueous solution of choline hydrogen carbonate was reacted with one equivalent of the corresponding acid at room temperature. All the obtained ILs exhibited good thermal stability, showing decomposition temperatures (T_d_, 50% weight loss) above 165 °C. Toxicity studies performed against different filamentous fungi of *Penicillium* genus, a validated model of eukaryotic organisms with the ability to grow in the presence of ILs, highlighted an increase in toxicity with increasing linear chain length. This side-chain length effect would be related to an increase in anion lipophilicity. In addition, the branched isomers of butanoate and pentanoate displayed lower grow inhibitory and fungicidal effects than the corresponding linear analogues. That is, branching, which usually decreases lipophilicity, also appears to reduce toxicity. The authors also carried out a biodegradability study with results of limited relevance due to the wide range of IL concentrations used. However, this study demonstrated that the toxic effects of cholinium alkanoates are lower than those of corresponding sodium alkanoates. Additional biodegradability studies performed by J. Pernak et al. [69] on FAILs containing tetralkylammonium cations confirmed that most of them are readily biodegradable. The authors appreciated the significant influence of both cationic and anionic alkyl substituents on biodegradability. Thus, FAILs with alkyltrimetylammonium cations and unsaturated alkyl anions displayed better biodegradability properties than those containing dialkyldimethylammonium cations and saturated alkyl anions.

To obtain further insight into the cholinium-based ILs biocompatibility and the effect of anions on this property, J. Coutinho et al. [80] conducted ecotoxicity studies based on the luminescence response of the marine bacteria *Vibrio fischeri* using standard Microtox^®^ liquid-phase assays. The tested ILs include different cholinium derivatives, some displaying aliphatic carboxylate anions such as acetate, propanoate or butanoate. The reported results categorize these compounds as nearly nontoxic against *V. fischeri*, with EC_50_ values at 30 min of exposure time ranging from 487.90 mg/L to 884.10 mg/L. The detected toxicity order was as follows: [Ch][But] < [Ch][Ac] < [Ch][Prop], suggesting that anion identity contributes to toxicity, although this trend does not agree with the alkyl chain effect. In addition, the ecotoxicity of these cholinium carboxylate-based ILs in aquatic media was found to be lower than that of [Ch]Cl. Surprisingly, however, they display more aquatic toxicity than some chlorinated organic solvents, such as chloroform or dichloromethane.

The cholinium carboxylate-based ILs mentioned above were then studied by the same authors in three standard aquatic biological models, the microalga *Raphidocelis subcapitata*, the macrophyte *Lemna minor*, and the freshwater cladoceran *Daphnia magna*, to confirm the anion role in ecotoxicity across different biological systems [68]. Considering the results obtained, it was not possible to establish structure-ecotoxicity relationships for these compounds due to the variability of response of the biological models. However, the findings confirmed that cholinium ILs are not completely devoid of toxicity and that the anion structure is a factor determinant of their ecotoxic behavior.

Subsequently, to clarify the anionic side-chain length effect on toxicity of FAILs, additional ecotoxicity and mammalian toxicity studies were conducted.

P. Mester et al. [81] performed ecotoxicological tests on FAILs containing anions with alkyl chain length ranging from C_1_ to C_18_ and different imidazolium cations, using multilevel biological assays that involved enzyme inhibition, virucidal and bactericidal tests. The results, which were compared with those described for the corresponding imidazolium chlorides, did not confirm a general anionic side-chain effect. The influence of anion chain length was only evident at the enzymatic level and in Gram-positive bacteria, whereas Gram-negative bacteria and viruses were unaffected by increases in anion chain length.

On the other hand, in their search for new ILs with low toxicity, A. Meirelles et al. [82] combined caprate (C_10_) and oleate (C_18_, unsaturated at C_9_) anions with the bis(2-hydroxyethyl)ammonium cation. The two proposed protic FAILs were successfully obtained as liquids at room temperature via a Brønsted acid-base reaction [83]. The authors conducted mammalian toxicological studies in Wistar rats to check subacute oral toxicity. Based on lipid peroxidation analyses of plasma, liver, and kidney samples, they concluded that no signs of toxicity were observed after 30 days of ingestion.

Taking the potential of ILs into account, all these findings represent an advance in the development of bio-based products, while also highlighting the need for further toxicological research aimed at assessing the environmental and human health impact of FAILs.

#### 2.1.2. Biological Potential of FAILs

**Figure 2:**
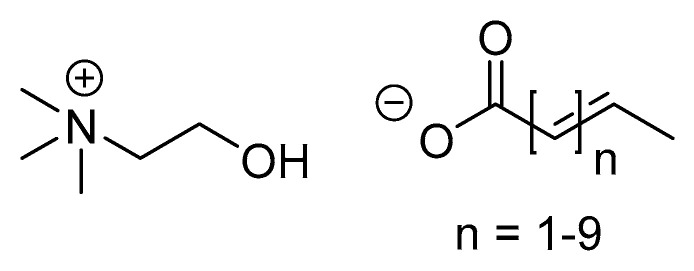
General structure of choline carboxylic acid-based ILs with antimicrobial activity.

The ability of ILs to interact with and modify cell membranes makes them very interesting for the development of antimicrobial agents. In the last few years, several ILs containing quaternary ammonium cations, particularly choline and other acyclic quaternary ammonium species, paired with carboxylate anions were proposed as potential bactericides effective against both Gram-positive and Gram-negative bacteria. In this context, E. Tanner et al. [48] described a series of choline carboxylic acid based-ILs obtained by the reaction of choline bicarbonate with several carboxylic acids at room temperature, providing both ILs and DESs. The ILs here described (Figure 2) consist of choline combined in an equimolar ratio with different carboxylate anions, varying in alkyl chain lengths (from four to twelve carbon atoms) and saturation degree. Their antibacterial activity was tested against *Escherichia coli* (Gram-negative) and methicillin-resistant *Staphylococcus aureus* (MRSA, Gram-positive) to explore the anion structure effect on bactericidal properties.

The authors concluded that the minimal bactericidal concentration (MBC) values for both types of bacteria decreased as the length of the anion alkyl chain increased, with ILs derived from saturated anions being the most effective. In addition, the bactericidal activity of these choline-based FAILs was superior to that of choline and the corresponding fatty acids individually. Human biocompatibility studies, on the other hand, revealed minimal cytotoxic effects against the HEK 293 cell line at the corresponding MBC values.

The combination of choline bicarbonate and geranic acid (3,7-dimethylocta-2,6-dienoic acid) led to several formulations collectively identified as CAGE [49], in which the two species were in different molar ratios, providing a true IL (1:1 CAGE) and some DESs (1:2, 1:4 and 2:1 CAGE), all of them stable under experimental conditions at 37 °C. CAGE IL showed a lower MBC against *E. coli* than choline or geranic acid alone, also revealing that the combination of both ions increases bactericidal properties. The authors used molecular dynamics simulations to explain the action mechanism of choline geranate on *E. coli* cell membrane and justify the disrupting effect detected via a choline attraction to the membrane to form stable ionic interactions followed by a geranate hydrophobic tail insertion. Moreover, the carboxylate group of geranate was also stabilized though interactions with the immersed choline residues.

Fatty acids and choline are two natural bioactive compounds also used as food additives. In this context, some choline-based FAILs and their ethanolamine analogues, such as cholinium stearate ([Ch][C_18_COO]) or diethanolammonium stearate ([H_2_EA][C_18_COO]) were described by A. Meirelles et al. [50] as highly efficient emulsifiers exhibiting good kinetic stability and preservative properties due to their antimicrobial activity against fungi and bacterial. The proposed ILs, which were easily synthesized from choline hydroxyde or diethanolamine and stearic acid, present notable technical advantages over common emulsifiers by combining both emulsifying and antimicrobial effects within a single formulation. Likewise, C. Freire et al. [84] proposed combining mixtures of choline hexanotate ([Ch][Hex]) and choline citrate ([Ch][Cit]) with polysaccharides to produce transparent, antibacterial and biocompatible films for biomedical and food packaging applications.

J. Pernak et al. described some tetraalkylammonium ILs with carboxylate anions derived from naturally occurring triglycerides or vegetable oils as potential antimicrobials, feeding deterrents and readily biodegradable [69]. The salts studied were successfully obtained by hydrolyzing selected triglycerides (glyceryl tristearate or glyceryl trioleate) or vegetable oils (canola or coconut oil) with different quaternary ammonium hydroxides. It is worth noting that in some of these salts, specifically those obtained from vegetable oils, the anion consists of a mixture originating from different fatty acids.

**Figure 3:**
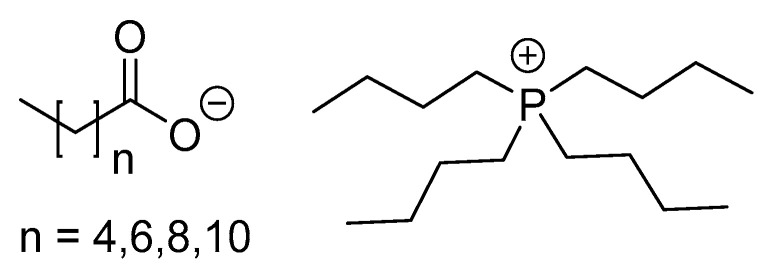
General structure of tetrabutylphosphonium FAILs with antimicrobial activity.

FAILS containing a quaternary phosphonium cation (Figure 3) were also described by M. Vraneš et al. [85] as potential antimicrobials against Gram-negative and Gram-positive bacteria, yeasts and fungi.

The four FAILs described were readily synthesized by a neutralization reaction between tetrabutylphosphonium hydroxide [TBP][OH] and the appropriate fatty acid and exhibited activity against all tested microorganisms. They were more effective against Gram-positive bacteria than against Gram-negative bacteria, displaying MBC values ranging from 0.60 to 17.5 mmol/L and from 2.50 to 17.5 mmol/L, respectively. This behavior is consistent with the highly organized structure of Gram-negative bacterial cell membrane. It should be noted that the antimicrobial properties of the fatty acids used as starting materials were enhanced upon their conversion into FAILs. In addition, increasing anionic alkyl chain was found to enhance antibacterial potency. However, the influence of side-chain length was less evident in assays involving yeasts and fungi.

**Scheme 3:**
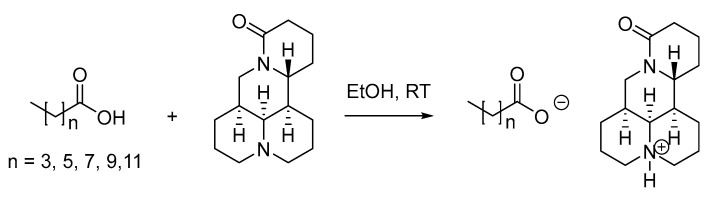
Synthesis of FAILs containing the matrinium cation.

FAILs derived from bioactive alkaloids or classical drugs were also explored by J. Zhang et al. as a new cost-effective chemotherapeutic approach [86]. For example, matrine, an alkaloid isolated from plants of genus *Sophora* with an extensive pharmacological profile, was converted, through an acid-base neutralization, into a series of matrinium-base ILs containing different long-chain fatty acid anions (Scheme 3). The target room-temperature FAILs exhibited enhanced antibacterial and anticancer activities compared with matrine itself. Both properties increased with the anion alkyl chain elongation, confirming the influence of the anion side-chain length on biological activity.

The mixture of the above-mentioned FAILs was further described by the same authors as an environmentally friendly matrine-based IL composed of coconut fatty acids, named [Mat][Coc]. This IL exhibited excellent antioxidant and antibacterial properties and was proposed as a potential additive to enhance the preservation properties of food packaging films [87]. Moreover, [Mat][Coc] was also suggested as a carrier for the transdermal delivery of peptide APIs like conotoxins [88], offering a promising alternative to conventional injectable drug formulations.

**Figure 4:**
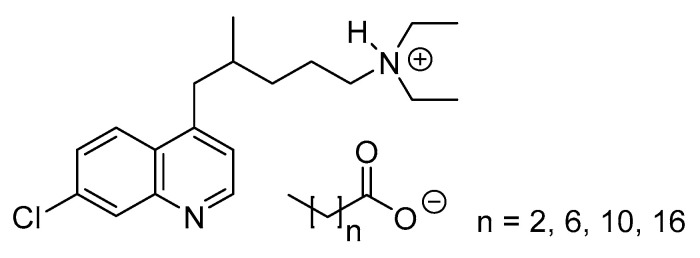
General structure of FAILs derived from chloroquine.

On the other hand, chloroquine, a well-known antimalarial drug that is inactive against some *Plasmodium falciparum* strains, was also used by P. Gomes et al. [74] to synthesize novel FAILs (Figure 4), which were subsequently evaluated in vitro against both chloroquine-sensitive and chloroquine-resistant *P. falciparum* strains.

All chloroquine-based FAILS studied exhibited stronger antimalarial activity than chloroquine phosphate, the conventional chloroquine formulation, with IC_50_ values in the nanomolar range. Among them, chloroquine laurate (dodecanoate) was the most potent FAIL of this series, with IC_50_ values of 4 and 110 nM against chloroquine-sensitive and chloroquine-resistant *P. falciparum* strains, respectively.

In addition, it is worth nothing that the chloroquine-based FAILs activity decreased slightly when the anionic alkyl chain was either shorter or longer than that of laurate. Furthermore, physicochemical analyses revealed that the enhanced activity of chloroquine in these systems is closely related to amphipathic nature and surface-active properties of the fatty acid carboxylate anions. Since lauric acid itself lacks intrinsic antiplasmodial effects, the authors concluded that the laurate anion in chloroquine-based FAILs provides the optimal chain length for the activity.

#### 2.1.3. Physicochemical Properties of Bioactive FAILs

The analysis of the physical properties of choline-carboxylate-based ILs reported by C. Silva-Pereira et al. [77] shows that they exhibit relatively low melting points, ranging from 80 °C for [Ch][Ac] to 26 °C for [Ch][Oct], allowing most of them to remain liquid at room temperature. In addition, a negative glass transition temperature was identified for [Ch][Prop] (T_g_ = −74 °C), indicating high molecular mobility in the amorphous state. These characteristics, together with onset degradation temperatures (T_onset_) between 97 °C and 169 °C and decomposition temperatures (T_d_) ranging from 166 °C to 210 °C, confirm good thermal stability suitable for moderate-temperature operations and various industrial applications.

This study is complemented by an analysis of the effect of temperature on the thermophysical properties of selected FAILs, including [Ch][Prop], [Ch][But], and [Ch][Hex], conducted by N. Muhammad et al. [89]. For example, density (ρ) decreases linearly with temperature, ranging from 1.0746 g·cm^−3^ at 293.15 K to 1.0401 g·cm^−3^ at 353.15 K for [Ch][Prop], while the refractive index (n^D^) follows a similar trend, decreasing from 1.4705 at 293.15 K to 1.4555 at 333.15 K in the case of [Ch][Prop]. Likewise, [Ch][But] and [Ch][Hex] showed slightly higher values, reaching 1.4716 and 1.4726 at 293.15 K, respectively. These variations reflect thermal expansion, quantified by the isobaric thermal expansion coefficient (α_p_), which remains around 5.32·10^−4^ K^−1^ for [Ch][Prop] at 293.15 K and 5.85·10^−4^ K^−1^ for [Ch][Hex] at 353.15 K, which tends to increase slightly with longer anion chains.

Dynamic viscosity (η) is the most notable property due to its magnitude and thermal sensitivity. At 293.15 K, [Ch][Prop] exhibits 395.8 mPaּּ·s, [Ch][But] 833.1 mPa·s, and [Ch][Hex] 929.2 mPa·s, with values decreasing significantly as temperature rises, reaching 25.4 mPa·s for [Ch][Prop] at 353.15 K. This behavior follows a Vogel-Fulcher-Tammann-type relationship, typical of ILs, and indicates a strong dependence of ionic mobility on temperature as well as the influence of Coulombic interactions and hydrogen-bonding networks.

Other properties related to density and calculated at 298.15 K, such as molecular vo-lume (V^M^), standard entropy (S^0^), and lattice energy (U_pot_), confirm that these ILs possess relatively low lattice energies, ranging from 434 kJ·mol^−1^ to 465 kJ·mol^−1^, and high entropies of up to 475.7 J·K^−1^·mol^−1^. These values reflect a high degree of molecular disorder, explaining their low melting points and relative stability. Such parameters are particularly relevant for understanding their behavior as solvents and their ability to interact with biomolecules.

The physical properties studied by both research groups [77,89], including melting point, glass transition, thermal stability, density, viscosity, refractive index, and derived parameters, confirm that converting carboxylic acids into choline-based ILs reduces volatility, improves chemical stability, and introduces structural features that govern macroscopic behavior. These characteristics are essential for applications in green chemistry, particularly in selective extraction processes and the formulation of aqueous biphasic system (ABS), where high polarity, low vapor pressure, and moderate thermal stability are essential for technical feasibility [77]. Furthermore, high viscosity and specific solute-interaction capacity, together with parameters such as density, refractive index, and lattice energy, support their use in biopolymer dissolution, and enzymatic processes, thereby expanding their potential in biotechnological applications.

A recent study conducted by J. Wei et al. [90] provided a detailed thermophysical characterization of the homologous series of choline–carboxylate ILs containing short-chain anions (formate, acetate, propionate and butyrate), offering further insight into the behavior of carboxylate-based systems. Their data reveal systematic decreases in density and surface tension, together with increasing ionic conductivity with rising temperature and anion chain length. A molecular interpretation of this behavior highlights the dominant role of O–H–··O hydrogen-bonding between the choline hydroxyl group and the carboxylate anion, along with the increased conformational freedom of longer anions. These results complement previous findings [77,89], confirming that both microscopic interactions and anion mobility govern macroscopic properties such as ionic transport, surface activity and packing efficiency.

C. Freire et al. [84] reported detailed thermophysical data for [Ch][Hex] and choli-nium citrate ([Ch][Cit]), two ILs selected for their antimicrobial activity and subsequent incorporation into polysaccharide matrices. [Ch][Hex] exhibits a melting point (T_m_) of 52 °C, an onset of degradation (T_onset_) at 106 °C, and a temperature corresponding to 50% mass loss (T_d_) of 169 °C. In contrast, [Ch][Cit] shows higher values, T_m_ = 103 °C and T_d_ = 215 °C, reflecting the influence of anion structure on thermal stability. The citrate anion, due to its bulkier structure and multiple functional groups, provides greater thermal resistance than the hexanoate anion; however, both choline-based ILs reduce the overall thermal stability of the films compared with pure polysaccharides.

In addition to cholinium-based systems, other families of carboxylate ILs derived from fatty acids and natural oils have been investigated to evaluate how structural variations in cation and anion influence melting behavior, thermal stability, solubility, and functional performance.

The physical state at room temperature of the quaternary ammonium ILs studied by J. Pernak et al. [69], which contain anions derived from triglycerides and mixtures of anions from vegetable oils as canola (CAN) and coconut oil (COCO), depends on both cation architecture and anion saturation. Salts containing the didecyldimethylammonium (DDA) cation and saturated anions as stearate (ST) form waxes, whereas hexadecyltrimethylammonium (CET) cation combined with unsaturated anions as oleate (OL) yields viscous liquids. Phase transition data show T_m_ below 81 °C for most compounds, with [CET][OL] melting at 38 °C and [CET][COCO] exhibiting multiple melting fractions at −21 °C, 11 °C and 36 °C, reflecting structural heterogeneity. Thermal stability remains high, with T_onset_ between 180 and 212 °C and T_d_ of up to 290 °C, comparable to the tetrabutylammonium (TBA)-based systems later reported by R. Gusain and O. Khatri [91]. Thermogravimetric analysis of [TBA][OL] confirms high thermal stability, with a T_d_ of 268.4 °C. DSC data show a T_m_ of −15.3 °C and a crystallization temperature (T_c_) of −26.2 °C.

Other studies on ammonium-based ILs [92,93] demonstrated that FAIL density and viscosity decrease with increasing temperature, while anion chain length and branching significantly increase viscosity and reduce ionic mobility. In addition, R. Belchior-Torres et al. [94] confirmed the high miscibility of *n*-butylammonium oleate with alcohols (propanol, butanol, pentanol, hexanol) across the entire composition range, indicating a strong affinity for polar organic media. This characteristic makes it an excellent candidate for biocatalytic processes in ionic environments and for biomolecule extraction, where compatibility with proteins and enzymes is essential.

M. Vraneš et al. [85] in their study about ILs based on the tetrabutylphosphonium (TBP) cation and medium-chain fatty acids anions such as, hexanoate, octanoate, decanoate, and dodecanoate (Figure 3), analyzing the influence of anion alkyl chain length on thermal, physical, and transport properties. TGA and DSC analyses revealed a single-step decomposition for all ionic liquids. The T_onset_ decreased with increasing anion chain length, ranging from 326.7 °C for [TBP][Hex] to 240.1 °C for [TBP][Dodec]. Similarly, the maximum degradation temperature (T_p_) varied between 354.6 °C and 297.2 °C for the same compounds. No melting points were detected within the studied temperature range, confirming that these ILs remain in the liquid state over a wide thermal window. Glass transition temperatures (T_g_) were observed between 73.0 °C and 79.2 °C, showing a slight increase with increasing chain length. Density measurements revealed a systematic decrease with both temperature and anion chain length. At 293.15 K, density values ranged from 0.92222 g·cm^−3^ for [TBP][Hex] to 0.90602 g·cm^−3^ for [TBP][Dodec], reflecting reduced packing efficiency and increased free volume as chain length grows. Viscosity also exhibited a marked dependence on temperature and chain length; at 293.15 K, values ranged from 483.06 mPa·s for [TBP][Hex] to 542.28 mPa·s for [TBP][Dodec], decreasing significantly at 313.15 K to 131.81 mPa·s and 152.37 mPa·s, respectively. This trend follows Arrhenius behavior, with activation energies for viscous flow between 48.39 kJ·mol^−1^ and 49.41 kJ·mol^−1^. Flow curves confirm Newtonian behavior across the entire shear rate range. Conductivity data and calculated molar conductivities indicated that conductivity increases with temperature but decreases with anion alkyl chain length. At 293.15 K, conductivity values range from 0.087716 mS·cm^−1^ for [TBP][Hex] to 0.045095 mS·cm^−1^ for [TBP][Dodec]. Activation energies for conduction are similar for all salts, between 43 kJ·mol^−1^ and 46 kJ·mol^−1^, suggesting comparable charge transport mechanisms among the ILs. Overall, ILs with longer alkyl chains exhibit lower density and conductivity, higher viscosity, and reduced thermal stability. These characteristics are attributed to enhance van der Waals interactions and the formation of hydrophobic domains, which reduce ionic mobility and increase flow resistance.

J. Zhang et al. [86] studied a family of ILs based on the matrinium cation (Mat) and anions derived from long-chain fatty acids (Scheme 3). This combination confers unique characteristics on these compounds, distinguishing them from conventional ILs both in their thermal behavior and in their physicochemical properties.

From a thermal perspective, all these compounds qualify as RTILs, exhibiting transition temperatures (T_g_/T_m_) below −31 °C. This behavior contrasts with that of analogous cholinium-based ILs, which tend to be solid under comparable conditions and display less thermal stability [77]. Accordingly, the T_d_ values of matrinium-based ILs range from 207.6 °C to 231.6 °C. This level of thermal stability is essential for applications involving elevated temperatures, as ILs do not possess a conventional boiling point and instead undergo thermal decomposition at high temperatures.

Regarding rheological properties, the viscosity measured at 25 °C ranges from 489.5 mPa·s to 325.5 mPa·s, showing a significant decrease with increasing alkyl chain length, which is unusual in ILs. A similar trend is observed for density, which ranges from 1.07 gּ·cm^−3^ to 1.00 gּ·cm^−3^, and for dipolarity/polarizability (π*), with values decreasing from 0.78 to 0.67. In contrast, hydrogen-bond basicity (β) increases from 0.15 to 0.69 when the anion chain length grows. These results indicate that the matrine-based ILs are less polar than conventional ammonium ILs, favoring interactions with lipophilic compounds and suggesting potential applications in the extraction and dissolution of weakly polar molecules. Additionally, the increase in β with longer anion chains reflects enhanced hydrogen-bond accepting capacity, which can be attributable to the decreasing acidity of the corresponding fatty acid.

The RTILs derived from the antimalarial drug chloroquine (CQ), [CQ][butyrate], [CQ][octanoate], [CQ][laurate], [CQ][myristate], and [CQ][oleate] (Figure 4), studied by P. Gomes et al. [74] exhibited one or two thermal degradation events within the temperature range of 93 °C to 236 °C, depending on the alkyl chain length. Specifically, [CQ][butyrate] showed degradation at 93.2 °C and 236.9 °C, [CQ][octanoate] at 120.7 °C and 220.5 °C, [CQ][laurate] at 156.9 °C and 228.5 °C, while [CQ][myristate] displayed a single degradation event at 227.7 °C, and [CQ][oleate] at 197.1 °C. These values are lower than that of commercial chloroquine phosphate (301.7 °C) but remain sufficiently high to ensure stability under ambient conditions and pharmaceutical formulation requirements. The RTILs derived from chloroquine and fatty acids act as surface-active ILs (SAILs), exhibiting significant interfacial activity. Notably, [CQ][laurate], which displayed the highest antimalarial activity, reduced the surface tension of water from 72.0 mN·m^−1^ to 29.8 mN·m^−1^. Furthermore, in mixtures with cetyltrimethylammonium bromide (CTAB), [CQ][laurate] decreased the CMC from 0.84 mmol·kg^−1^, observed for pure CTAB, to 0.057 mmol·kg^−1^, with a surface tension at CMC of 22.0 mN·m^−1^.

### 2.2. Phenolic Acid-Based ILs

Natural phenolic acids are secondary metabolites of plants that stand out for their antioxidant and anti-inflammatory properties [65]. They contain one or more aromatic rings directly bonded to hydroxyl groups, an ideal structure for scavenging free radicals and reducing oxidative damage, effects associated with protection against cancer development and various inflammatory diseases. These compounds are classified as hydroxybenzoic acids or hydroxycinnamic acids and can occur either in free form or conjugated with other molecules. One of the major drawbacks of phenolic acids for inclusion in pharmaceutical formulations is their low water solubility, which can lead to issues with bioavailability. Converting these molecules into API-Ls has emerged as a promising strategy to overcome this limitation [95] and, from a more general perspective, may contribute to relaunching drugs with unfavorable pharmacokinetic profiles.

#### 2.2.1. Biological Potential of Phenolic Acid-Based ILs

**Figure 5:**
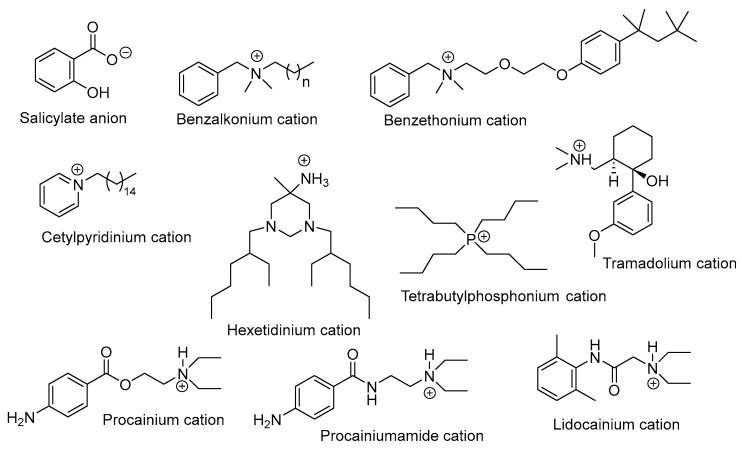
Structure of salicylate anion and of several active ammonium cations used to provide potential API-ILs.

Salicylic acid (2-hydroxybenzoic acid), a natural phenolic acid with poor water solubility, is an active metabolite of acetyl salicylic acid, a well-known non-steroidal anti-inflammatory drug. Systemic administration of salicylic acid causes serious gastrointestinal disorders, which is why its current use is restricted to topical applications in various medical and cosmetic formulations. R. Rogers et al. [51] synthesized several ILs to regulate the solubility, improve the intestinal tolerability, and reduce the side effects of salicylic acid. Several cations with different pharmacological properties, including antimicrobial (benzalkonium, benzethonium, cetylpyridinium, hexetidinium and tertrabutylphosphonium), analgesic (tramadolium) or local anesthetic activity (procainium, procainiumamide and lidocainium) were combined with the salicylic acid anion, providing dual API-ILs with new potential therapeutic profiles (Figure 5).

Most salicylate ILs were obtained in good yield by ionic metathesis reactions performed in aqueous medium. Some were also successfully prepared under free-solvent conditions by melting stoichiometric mixtures of the acid with the corresponding base. Regarding their thermal properties, all synthesized salicylates, except hexetidinium salicylate (mp 106.81 °C) and tramadolium salicylate (mp 176.17 °C) were low-melting solids or liquids at room temperature, and they displayed greater thermal stability than the sa-licylic acid, its alkali salts and even the free bases or salts of the corresponding cations. In addition, the salicylate ILs were described to be more stable against moisture and temperature than the corresponding acetyl salicylate ILs, since the acetylsalicylate anion is highly sensitive to hydrolysis. This confirms the importance of ion stability in obtaining stable liquid salts. Although no biological studies were performed, the authors proposed that salicylate ILs containing antibacterial cations could serve as potential antimicrobials and biocides.

Subsequently, M. Saraiva et al. [52] performed experimental studies on some aspects related to pharmacokinetics and pharmacodynamics of these compounds, such as plasma protein-binding affinity, partition coefficient and surfactant properties, using cetylpyridinium salicylate ([CetPy][Sal]) and benzalkonium salicylate ([Be][Sal) (Figure 5). The two compounds showed good affinity for human serum albumin and good antimicrobial potential, as they retained the surfactant properties of the parent cations and demonstrated improved partitioning into the lipid phase of biological membranes compared with the original inorganic salts. These results revealed a favorable pharmaceutical profile for both salicylate ILs, suggesting their suitability for transdermal or topical drug formulations.

**Figure 6:**
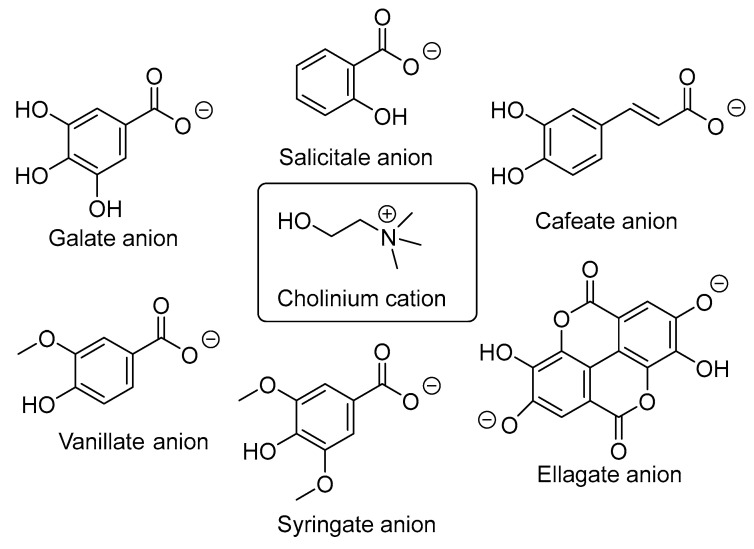
Several phenolic acid anions used to provide cholinium-based salts as water soluble antioxidants.

The combination of the cholinium cation with the salicylate anion yielded the pioneering API-IL, [Ch][Sal], which exhibited analgesic, antipyretic, and anti-inflammatory properties and better water solubility than salicylic acid [96]. [Ch][Sal] has already been incorporated into some pharmaceutical formulations [95]. In this context, M. Freire et al. [97] reported a series of cholinium-based salts containing various phenolic acid anions, including caffeate, syringate, vanillate, ellagate, and gallate. These [Ch][Sal] analogues (Figure 6), synthesized by a neutralization reaction of [Ch][OH] with the corresponding acid, exhibited greater water solubility than their parent phenolic acids while retaining, or even enhancing, their antioxidant and anti-inflammatory properties. However, despite being composed entirely of ions, their melting points typically exceed 150 °C [97], and they therefore cannot be classified as ILs. Nevertheless, a few years later some of these salts as [Ch][Caf], [Ch]_2_[Ell] and [Ch][Gal] are incorrectly referred to as ILs [54,98].

**Figure 7:**
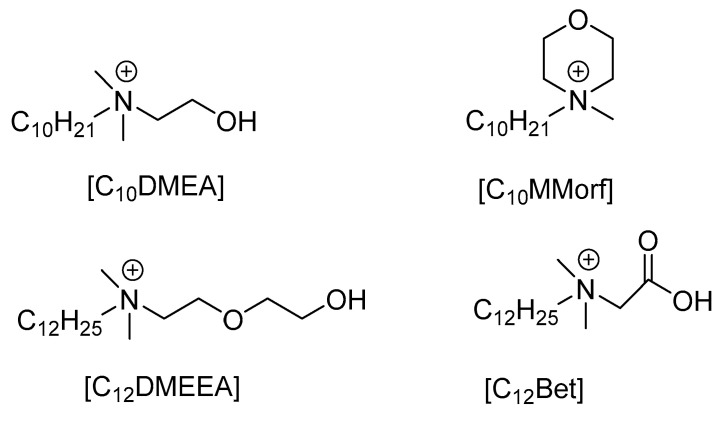
Quaternary ammonium cations combined with gallate anion.

The antioxidant activity of phenolic acids is largely governed by both the number and the positional arrangement of their phenolic hydroxyl groups. Accordingly, dihydroxy- and trihydroxybenzoic acids exhibit stronger antioxidant activity than their mo-nohydroxylated couterparts. Gallic acid stands out as one of the most effective radical scavengers due to its polyphenolic structure, and many current antioxidant formulations contain gallic esters. To overcome the limited water solubility and improve the biological activity of gallic acid and derivatives, K. Czerniak et al. proposed their conversion into multifunctional ILs [99]. On their approach the gallate anion was combined with various quaternary ammonium cations bearing a long alkyl chain (Figure 7).

The target compounds were obtained from the adequate alkyl ammonium bromides in two steps: First, conversion into the corresponding ammonium hydroxides by using an anion-exchange resin in methanol, followed by neutralization with gallic acid. In contrast, preparation of the salt containing the amphoteric betaine cation required only a single step, involving protonation of the amino acid carboxylate with gallic acid in methanol. All the resulting salts were liquid or waxy solids at room temperature and exhibited high thermal stability, good surface activity, and markedly higher water solubility than gallic acid, indicating that the presence of a long alkyl chain does not seem to affect solubility when highly hydrophilic groups are present. The antioxidant properties of these gallate-based ILs, measured using the DPPH (2,2-diphenyl-1-picrylhydrazyl) radical scavenging assay, were comparable to those of gallic acid (IC_50_ = 5.53 ± 0.15 µM) or even slightly superior. No negative effects of the alkyl chain were observed. In fact, [C_12_Bet][Gal] and [C_12_DMEEA][Gal], both with a dodecyl chain, were the most effective, with IC_50_ values of 4.82 ± 0.19 μM and 4.69 ± 0.15 μM, respectively. These two gallate-based ILs showed antimicrobial activity comparable to that of gallic acid, against both Gram-positive and Gram-negative bacteria, although their potency was lower than that of conventional quaternary ammonium salts, such as benzalkonium chloride and didecyldimethylammonium chloride.

Protocatechuic acid (PCA) and gentisic acid (GA) are two naturally occurring dihydroxybenzoic acids that, in addition to their antioxidant activity, exhibit a wide variety of biological effects. Both acids were used as anions to obtain antioxidant dicationic ILs (DILs) [100].

DILs represent a new class of ILs featuring multivalent cations, in which two or more cationic head groups are connected by an alkyl chain. DILs often show better surface activity than conventional ILs [101], which makes them interesting for applications such as antioxidants and chemotherapeutic agents.

**Figure 8:**
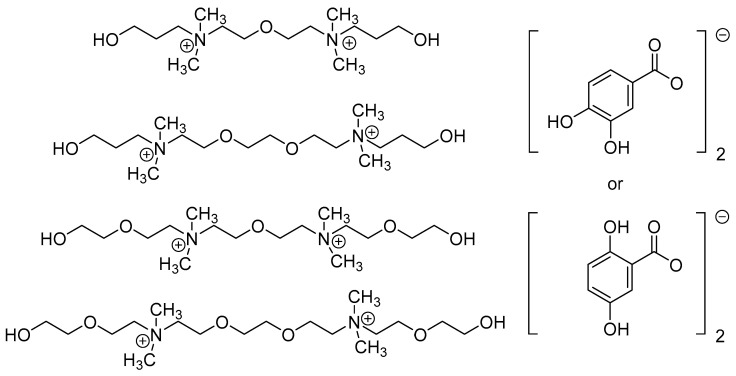
DILs containing different bisammonium cations combined with PCA or GA anions.

Eight DILs (Figure 8) were successfully synthesized by K. Czerniak and F. Walkiewicz [100] via an acid-base neutralization reaction between four distinct quaternary bis(ammonium) dihydroxides and two naturally occurring phenolic acids, namely PCA (3,4-dihydroxybenzoic acid) and GA (2,5-dihydroxybenzoic acid). All resulting compounds exhibited high aqueous solubility and good thermal stability. In vitro antioxidant studies performed using two spectrophotometric assays, the DPPH radical and ABT radical cation scavenging methods, demonstrated that these DILs possessed markedly higher activity than the corresponding free acids. This enhancement was attributed to the presence of two hydroxybenzoate anions within the molecular structure. Furthermore, the investigated DILs displayed antioxidant properties comparable to those previously reported for monocationic gallate-based ionic liquids [99], and variations in the spacer length did not appear to significantly affect their antioxidant efficacy.

**Figure 9:**
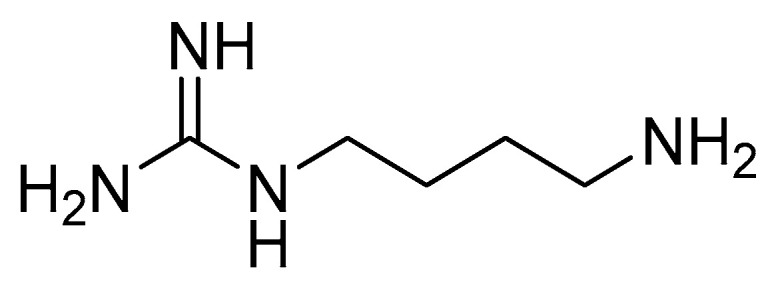
Chemical structure of agmatine.

Agmatine is an endogenous aminoalkylguanidine derived from arginine decarboxylation (Figure 9). It is widely distributed in peripheral tissues and the central nervous system, where it would function as a neurotransmitter. This potentially dicationic species was combined by M. Vranĕs et al. with different carboxylate anions, including salicylate, to provide biocompatible DILs as possible carriers of APIs [102].

**Figure 10:**
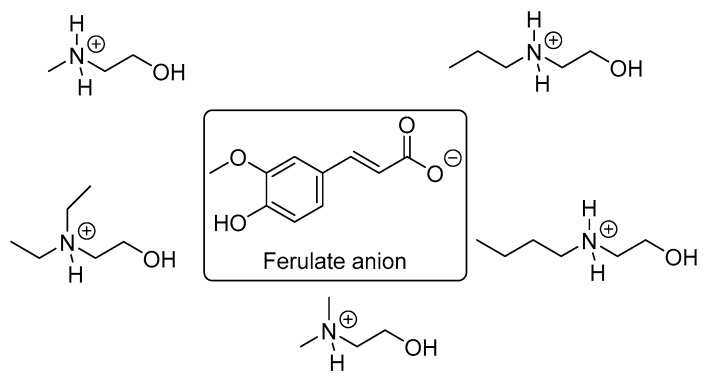
Ferulate anion and ethanolammonium cations components of bioactive protic ILs.

Hydroxycinnamic acids and their analogues display the characteristic bioactive properties of phenolic compounds, which are attributed not only to the presence of hydroxyl groups but also to their conjugated double-bonded structures. Several hydroxycinnamate-based ILs containing alkanolamnonium cations have been explored as promising bioactive agents, and specifically as free-radical scavengers [103,104]. Some of these ILs are protic ILs [103], obtained by reacting equimolecular amounts of ferulic acid (*E*-4-hydroxy-3-methoxycinnamic acid) and alkanolamines bearing different alkyl chain (Figure 10).

Most of the resulting ammonium ferulates are liquids at room temperature (RTILs) and exhibit moderate thermostability. The number of alkyl groups on the nitrogen contributes favorably to this stability. All these ILs demonstrate improved solubility in polar solvents and enhanced antioxidant activity compared with ferulic acid (IC_50_ = 21.40 ± 0.05 µM), with IC_50_ values ranging from 12.93 ± 0.05 to 17.40 ± 0.04 μM. In addition, the ferulates derived from tertiary alkanolamines exhibited the best radical scavenging properties.

**Figure 11:**
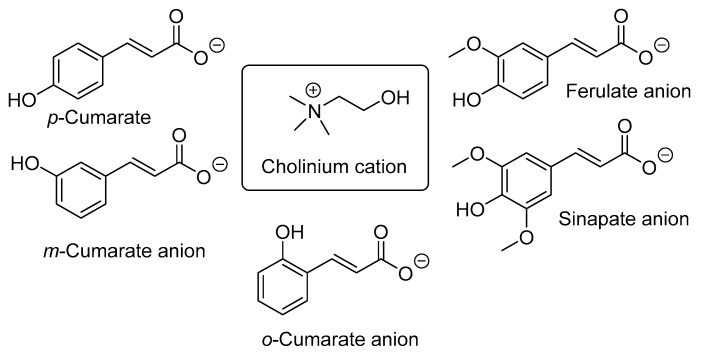
Cholinium cation and different hydroxycinnamate anions components of bioactive ILs.

Ferulic acid and other hydroxycinnamic acids, including sinapic, *o*-coumaric, *m*-coumaric, and *p*-coumaric acids, were successfully converted into the corresponding cholinium-based ILs by F. Mocci et al. through neutralization of choline hydroxide with a slight excess of the respective carboxylic acid [104]. The resulting compounds (Figure 11), in contrast to choline caffeate, an analogous ionic salt previously reported [97], are RTILs. These RTILs exhibit enhanced aqueous solubility, slightly lower thermal stability, and superior in vitro free-radical scavenging activity compared with their precursor carboxylic acids. Nevertheless, their overall antioxidant efficacy is lower than that of choline caffeate (IC_50_ = 33.4 ± 0.1 µM), which the authors attributed to electronic effects arising from the additional phenolic hydroxyl group in the caffeate anion. In addition, the negligible cytotoxic effects observed in mouse cell lines, both tumor and embryonic, points out their good biocompatibility. All these results support the potential of the proposed cholinium RTILs as alternatives to hydroxycinnamic acids in pharmaceutical applications.

**Figure 12:**
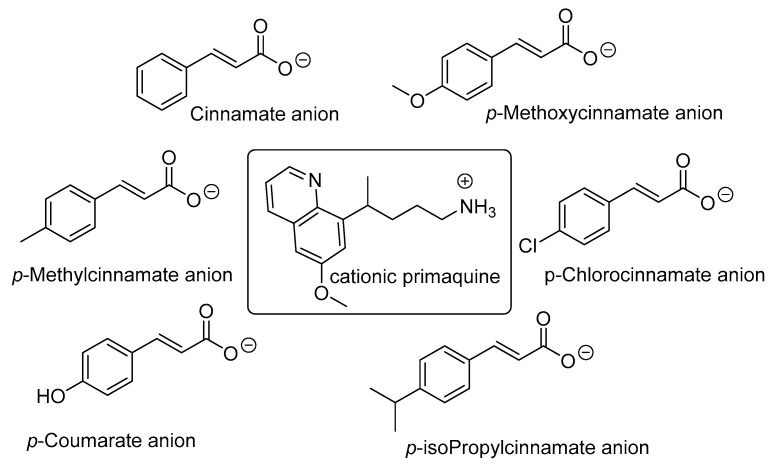
Cationic primaquine and the different cinnamate anions components of novel API-ILs.

On the other hand, several cinnamic acids, including *p*-coumaric acid, were employed by P. Gomes et al. [105] to synthesize protic ILs derived from primaquine (Figure 12), a classical antimalarial API known for its potent activity against the hepatic stages of different *Plasmodium* species, which are responsible for malaria relapses. However, primaquine exhibits limited efficacy against the erythrocytic stages of the parasite and is associated with certain toxicity concerns.

The target salts were obtained in high yields (88–99%) as viscous liquids through the neutralization of primaquine with an equimolar amount of the corresponding carboxylic acid [105]. The resulting compounds were subsequently evaluated in vitro against three developmental stages of the parasite: erythrocytic, hepatic, and gametocytic forms. These API-ILs demonstrated enhanced activity against the erythrocytic stages of both chloroquine sensitive and chloroquine resistant *Plasmodium falciparum* strains compared with primaquine and even surpassed certain N-cinnamoyl-primaquine covalent derivatives [106], while retaining their activity against hepatic stages at non-hepatotoxic concentrations. Furthermore, gametocytocidal assays revealed favorable outcomes, with efficacy comparable or superior to both N-cinnamoyl-primaquine analogues and primaquine itself. Therefore, replacing the covalent amide bond with an ammonium carboxylate ionic interaction increases activity against malaria erythrocytic forms, with the ionic formulations of primaquine with *p*-methoxycinnamic or *p*-isopropylcinnamic acid emerging as the most promising triple-stage antimalarial hits of this series. Subsequent studies in zwitterionic and anionic phospholipid membrane models performed by the same authors using differential scanning calorimetry (DSC) [107] revealed that primaquine-based ILs contribute to increased API permeation in malaria-infected erythrocytes, an effect that appears to be further enhanced by the lipophilicity of the cinnamate anion substituent.

#### 2.2.2. Physicochemical Properties of Bioactive Phenolic Acid-Based ILs

The thermal analysis of salicylate and acetylsalicylate-based ILs reveals notable differences in their physical behavior and thermal stability [51]. Overall, salicylate-derived ILs (Figure 5) tend to exhibit low glass transition temperatures (T_g_) and depressed melting points, favoring liquid or amorphous states at ambient temperature. Tetrabutylphosphonium salicylate, for example, shows a T_g_ of 56.5 °C and a T_m_ of 57.3 °C, together with markedly enhanced thermal stability, as evidenced by a T_onset_ of 307.9 °C, nearly twice that of pure salicylic acid (T_onset_ = 162 °C).

Other salicylate-based ILs, including cetylpyridinium and benzalkonium salicylates, exhibit moderate T_m_ (73.9–96.0 °C) while maintaining T_onset_ values above 170 °C, supporting their suitability for pharmaceutical applications. In contrast, benzethonium, lidocainium, procainium, and procainamidium salicylates do not display a well-defined melting point but present low T_g_ values (13–19 °C) combined with acceptable thermal stability (T_onset_ = 158–187 °C). These results demonstrate that the absence of a distinct melting transition does not necessarily correlate with reduced thermal robustness and supports the classification of these systems as stable glassy liquids.

Within the group of acetylsalicylate-based ILs, the trend is reversed, as these systems exhibit markedly lower T_g_ and T_m_ values than their salicylate analogues, accompanied by lower thermal stability. Cetylpyridinium acetylsalicylate is the only compound with a defined melting point (61.3 °C); however, its T_onset_ drops to 115.1 °C, far below that of its salicylate analogue (205.6 °C). R. Rogers et al. [51] attributed this behavior to the absence of hydrogen-bonding interactions in the acetylsalicylate anion, which diminishes structural cohesion and increases susceptibility to hydrolysis.

Consistent with these observations, salicylate-based ILs are thermally more stable, with T_onset_ values above 150 °C in all cases and reaching up to 307.9 °C for tetrabutylphosphonium salicylate. Moreover, their T_m_ or T_g_ remain compatible with pharmaceutical applications. In contrast, although acetylsalicylate-based ILs are more liquid-like and display very low T_g_ values, their reduced thermal stability and propensity for hydrolysis limits their long-term applicability.

Several years later, M. Saraiva et al. [52] evaluated the physicochemical properties of two of these salicylate-based ILs, cetylpyridinium salicylate ([CetPy][Sal]) and benzalkonium salicylate ([Be][Sal]). The study focused on key parameters with direct implications for stability, bioavailability, and membrane interaction, namely the CMC, the dissociation constant (K_d_, reflecting binding affinity to human serum albumin), and the partition coefficient (K_p_, an indicator of lipophilicity and biological membrane affinity).

Micelle formation, as described by the CMC, plays a crucial role in solubility, formulation stability, and interactions with biological membranes. The results showed that [CetPy][Sal] exhibits a significantly lower CMC than [Be][Sal] (0.19 mmol·L^−1^ versus 0.48 mmol·L^−1^), indicating that [CetPy][Sal] self-aggregates at lower concentrations. This behavior may enhance antimicrobial efficacy by facilitating interactions with bacterial membranes and reducing the amount of surfactant required to stabilize dispersed systems in topical formulations.

With respect to plasma protein binding, [CetPy][Sal] displayed a lower K_d_ value (34.5 mmol·L^−1^) compared to [Be][Sal] (74.5 mmol·L^−1^), indicating a stronger interaction with human serum albumin. This higher binding affinity may result in an increased bound fraction in plasma and could influence the release profile and bioavailability of the active compound.

Lipophilicity was assessed through the K_p_, determined using hexadecylphosphocholine (HDPC) micelles as biomimetic models of phospholipid bilayers. Both ILs exhibited a high affinity for hydrophobic environments; however, [Be][Sal] showed a higher K_p_ value than [CetPy][Sal], suggesting enhanced membrane partitioning. This characteristic may favor membrane penetration and could contribute to differences in antimicrobial performance, particularly in topical applications.

The study by M. Freire et al. [97] presents a comprehensive physical characterization of six cholinium-based salts with anions derived from natural phenolic acids, namely gallate, vanillate, syringate, ellagate, caffeate, and salicylate (Figure 6). Among these, only [Ch][Sal] displays the typical features of a pharmaceutical RTIL. [Chol][Sal] is liquid at ambient conditions (T_m_ = 38 °C), fully miscible with water, and thermally stable, with a T_d_ of 226 °C, ensuring safe handling and storage. Additionally, it exhibits a T_g_ of −56 °C and a cold crystallization (T_c_) of −14 °C, confirming its amorphous nature and liquid behavior at room temperature. This combination of properties, liquid state, complete aqueous miscibility, and high thermal stability, renders [Ch][Sal] particularly versatile for pharmaceutical and cosmetic applications, especially in liquid or gel formulations that require facile processing and rapid release of the active ingredient. In contrast, the remaining cholinium-based salts investigated in this study are crystalline solids with melting points well above 100 °C, which limits their applicability under similar conditions.

However, combining the gallate anion with long-alkyl-chain tetraalkylammonium cations, such as decyl(2-hydroxyethyl)dimethylammonium (C_10_DMEA), 4-decyl-4-methylmorpholinium (C_10_MMorf), dodecyl [2-(2-hydroxyethoxy)ethyl]dimethylammonium (C_12_DMEEA), or dodecyldimethylglycine (C_12_Bet), results in liquid or waxy solids at room temperature (Figure 7). The thermogravimetric analyses (TGA) performed by K. Czerniak et al. [99] revealed that these four ILs exhibit sufficient stability for biomedical applications, although slightly lower than that of pure gallic acid. Their T_onset_ ranged from 174 °C to 194 °C, while T_d_ occurred between 241 °C and 275 °C. These values are adequate to withstand sterilization processes (≤135 °C), confirming their suitability for clinical environments. Regarding water solubility, all ILs showed substantial improvements compared to gallic acid (11.00 ± 0.06 g·L^−1^). Specifically, [C_10_DMEA][Gal] reached 49.2 ± 1.8 g·L^−1^, [C_10_MMorf][Gal] 33.9 ± 2.2 g·L^−1^, [C_12_DMEEA][Gal] was completely miscible in water, and [C_12_Bet][Gal] exhibited a solubility of 20.8 ± 0.7 g·L^−1^. This improvement is attributed to the presence of polar functional groups in the cations, which promote favorable interaction with the aqueous medium. Furthermore, octanol/water partition coefficients remained low (0.07–0.42), indicating low hydrophobicity and a reduced risk of bioaccumulation.

K. Czerniak and F. Walkiewicz [100] described eight dicationic ILs (DILs) derived from two natural antioxidant anions, protocatechuate (PCA) and gentisate (GA), combined with different quaternary bis(ammonium) cations displaying ether spacers (Figure 8). All DILs exhibited T_g_ values rather than melting points, confirming amorphous character. For PCA-based ILs, T_g_ values ranged from 40.75 °C to 9.73 °C and decreased with increasing ether spacer length. In contrast, GA-based ILs showed T_g_ values between 1.70 °C and 8.64 °C, which was attributed to positional differences in hydroxyl groups.

Regarding thermal stability, the T_onset_ for PCA ILs ranged from 178 °C to 204 °C, whereas GA-based ILs exhibited higher stability, with T_onset_ values between 240 °C and 247 °C. The T_d_ ranged from 238 °C to 287 °C, and the peak decomposition (T_p_) occurred between 273 °C and 286 °C. Compared with the free acids (PCA: T_p_ = 262 °C; GA: T_p_ = 270 °C), the GA-based DILs demonstrated superior thermal stability, decomposing in a single step over a broad range (up to 120 °C), in contrast to the two-step degradation observed for the free acids. Solubility measurements at 25 °C revealed exceptional water solubility (>100 g·L^−1^) for all DILs, far exceeding that of PCA (29.4 g·L^−1^) and GA (22 g·L^−1^). The presence of hydroxyl-functionalized cations and ether linkages enhanced hydrophilicity, resulting in complete miscibility in water, methanol, and DMSO. Conversely, the studied ILs were insoluble in low-polarity solvents such as chloroform and hexane and showed negligible solubility in acetone or acetonitrile.

Vraneš et al. [102] characterized biocompatible DILs based on agmatine (Agm), a neurotranmiter of aninoguanidine estructure (Figure 9), combined with different anions derived from APIs, some of them of natural origin, such as salicylate and nicotinate (Nic). Both compounds exhibit an apparently viscous liquid state at room temperature, with no tendency to crystallize, supporting their classification as ILs. However, the DSC measurements revealed values of T_m_ above of 100 °C, along with glass transitions around 20 °C. The authors attributed the inhibition of crystallization to slow ion diffusion and inefficient crystal nucleation during cooling, which leads to the formation of a glassy state. This behavior can be rationalized by the nature of the anions, as bulky aromatic species such as Sal and Nic reduce packing efficiency and promote π-π interactions, thereby limiting the formation of ordered crystalline networks and favoring liquid or glassy states. Additional thermal characterization data revealed T_onset_ values above 200 °C for both ILs, indicating sufficient thermal stability for pharmaceutical applications involving drying or sterilization processes.

The combination of high thermal stability (>200 °C), liquid state at room temperature, and glass transition temperatures around 20 °C positions these DILs as promising candidates for drug delivery systems and formulations requiring aqueous compatibility and safe handling.

Additionally, the transformation of ferulic acid, a natural occurring hydroxycinnamic acid, into protic ionic liquids (PILs) represents an effective strategy to improve the critical physicochemical properties of this well-known antioxidant.

In this regard, M. Kassim et al. [103] studied five PILs obtained by combining the ferulate anion with cations from different ethanolamines (Figure 10). One of the most significant findings is the pronounced increase in solubility in polar media resulting from PIL formation. Ferulic acid exhibited solubilities of 769.24 ± 0.01 mg·L^−1^ in water and 953.13 ± 0.02 mg·L^−1^ in methanol, whereas the synthesized PILs reached concentrations exceeding 2000 mg·L^−1^. Among them, PILs derived from primary ammonium cations exhibited the highest solubility in these media. The hydrophobicity and steric effects of the cation were identified as key factors governing this behavior. Thermal analysis by TGA and DSC revealed that these PILs decompose at temperatures between 106.3 °C and 118.1 °C, with PILs derived from tertiary ammonium cations exhibiting the highest thermal stability. Although these T_onset_ values are lower than those of conventional ionic liquids, they remain sufficient for pharmaceutical processes conditions (<135 °C).

With respect to thermal transition, a wide range of T_g_ was observed, spanning from 6.16 °C to −25.99 °C, while no melting points were detected, indicating an amorphous structure. This behavior is associated with ionic mobility, and higher ion mobility leads to lower T_g_ values. In this case, bulkier cations create greater free volume between cation and anion interactions, resulting in reduced structural rigidity and lower T_g_.

F. Mocci et al. [104] studied the physicochemical properties of five RTILs derived from choline (Cho) and different hydroxycinnamic acids, including ferulic (Fer), sinapic (Sin) and three isomers of coumaric acid, *p*-coumaric (*p*-Coum), *m*-coumaric (*m*-Coum), and *o*-coumaric (*o*-Coum) acids (Figure 11). The formation of the ionic pairs markedly enhances aqueous solubility, shifting from low millimolar values in the acids to significantly higher concentrations for the corresponding salts. For example, the ferulic acid exhibits a solubility of 0.0047 ± 0.0007 M, whereas [Cho][Fer] reaches 0.6 ± 0.1 M. This two-order-of-magnitude increase is attributed to the presence of the hydroxyethyl group in the choline cation, as well as to the reduction in intramolecular hydrogen bonding within the anion, which favors the interaction with the aqueous medium. Thermal characterization by TGA and DSC revealed substantial differences between the free acids and their corresponding ionic salts. The acids display high melting points, ranging from 172 °C to 221 °C, and decomposition temperatures (T_d_) ranging from 211 °C to 269 °C, indicating high thermal stability. Upon conversion into RTILs stability decreases, particularly for [Cho][Sin] and [Cho][Fer]; nevertheless, T_d_ values remain above 100 °C in all cases, which is sufficient for sterilization processes. The reduced stability observed for [Cho][Sin] and [Cho][Fer] was attributed to the presence of methoxy groups in the anionic species, which decrease structural cohesion and facilitate thermal decomposition.

The conversion of conventional drugs into ILs introduces a structural innovation that could redefine their pharmacokinetic profile. In this context, P. Gomes et al. [105] performed computational predictions of key physicochemical parameters of six antimalarial RTILs derived from primaquine (PQ) and various cinnamic acids (Figure 12). Their results indicated that the selection of the counterion allows the fine-tuning of lipophilicity and water-solubility.

In a complementary study, the same authors [107] investigated the interaction of two of these RTILs ([PQ][p-ClCinn] and [PQ][p-iPrCinn]) with different membrane models using DSC, employing multilamellar vesicles of 1,2-dipalmitoyl-rac-glycero-3-phosphocholine (DPPC), a zwitterionic lipid that mimics the membrane surface of non-infected mammalian erythrocytes, and 1,2-dipalmitoyl-sn-glycero-3-phospho-rac-(1-glycerol) (DPPG), an anionic lipid resembling the surface charge of protozoan membrane and parasite-infected erythrocytes. The results obtained revealed significant interactions of both API-ILs with both membrane models. In the absence of API-IL, DPPC exhibits a T_m_ of 41.5 °C with ΔH_m_ of 7.22 kJ·mol^−1^. Incorporation of [PQ][p-ClCinn] lowered T_m_ to 40.7 °C and increased ΔH_m_ to 9.60 kJ·mol^−1^, while [PQ][iPrCinm] provides T_m_ values of 40.2 °C and ΔH_m_ values 9.30 kJ·mol^−1^. In DPPG, the effects were similar but more pronounced, indicating stronger interactions with the anionic membrane model.

Both primaquine-derived ILs demonstrate that the incorporation of cinnamoyl fragments allows the precise tuning of key thermophysical properties, including lipid phase stability and membrane penetration capacity. These findings open new perspectives for the rational design of multistage antimalarials.

### 2.3. Hydroxy Acid-Based ILs

Hydroxy acids (HAs) of natural origin are generally regarded as non-toxic compounds with a wide range of cosmetic and therapeutic applications [108,109]. The HAs group includes the aryl hydroxy acids discussed in Section 2.2. of this review, as well as arylaliphatic, aliphatic, polihydroxy and terpenoid hydroxy acids, among others. Many of these compounds exhibit antimicrobial, antioxidant, anti-inflammatory or anticancer activities. Notably, in numerous cases, their derivatives demonstrate greater biological activity than the parent acids, which is often attributed to an optimized hydrophilicity-lipophilicity balance [109].

#### 2.3.1. Biological Potential of Hydroxy Acid-Based ILs

Mandelic acid (2-hydroxy-2-phenylacetic acid) is a chiral α-hydroxy acid found in amygdaline, a cyanogenic glycoside presents in some seeds such as bitter almonds. It displays low toxicity toward mammal cells and has been used as a urinary tract antiseptic [110].

**Figure 13:**
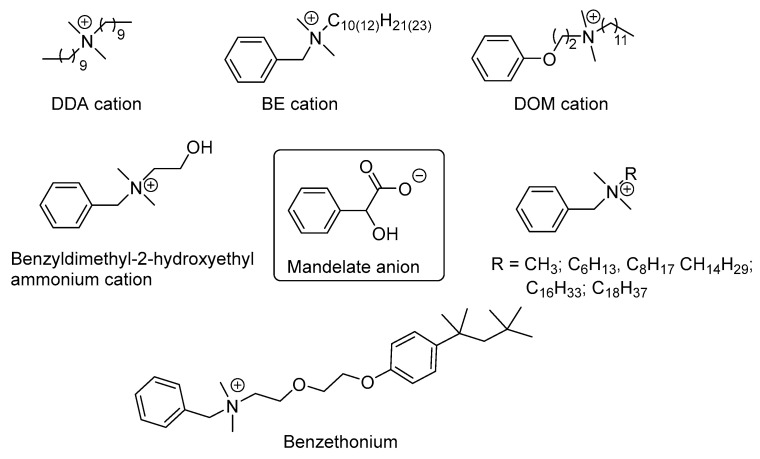
Several quaternary ammonium cations combined with mandelate anions and proposed as antimicrobial API-ILs.

The combination of mandelate with some quaternary ammonium species, including didecildimethylamonium (DDA), domiphen (DOM), benzalkonium (BE) and several benzylammonium cations, including benzetonium, was performed to improve the biocidal activity, reduce toxic and irritants effects associated with halide anions, and enhance druggability (Figure 13). The resulting quaternary ammonium salts, obtained in good yields (86–99%) via a metathesis reaction, were described as waxes or RTILs [111,112].

The availability of three mandelate forms (S-mandelate, R-mandelate and the racemic mixture) enabled the synthesis of chiral ILs based on DDA, DOM and BE. This approach allowed the comparison of the physicochemical and biological properties of the enantiomerically pure forms with those of their racemic counterparts [111]. No significant differences were detected between the optically active and racemic ILs. All compounds were stable under ambient conditions, water-soluble, and exhibited cation-dependent surface tension. An exception was noted for the T_g_, since four of the chiral ILs, specifically those containing DDA and BE cations, showed higher values than their racemic counterparts. According to the authors, this behavior may be attributed to more efficient molecular packing in certain enantiomerically pure forms.

In addition, all mandelate-based ILs exhibited activity against different bacterial and fungal species. In general, analogues with DDA and DOM cations were more potent than BE chloride, used as the reference drug. In contrast, ILs incorporating the BE cation displayed lower activity than the standard drug against both types of microorganisms. Notably, no differences in biological activity were detected between the two optically active forms of the nine mandelate ILs evaluated. However, significant differences between the R and S enantiomers were identified in terms of phytotoxicity [111].

Subsequently, several alkylbenzyldimethylammonium cations structurally related to BE, including benzyldimethyl-2-hydroxyethylammonium and benzetonium (Figure 13), were combined with the racemic mandelate anion. This strategy expanded the mandelate-based ILs series previously described by J. Pernak et al. [111], with the aim of establishing structure-activity relationships and evaluating cytotoxicity in mammalian cells [112].

According to this study, the newly synthesized mandelate-based ILs generally exhibited higher activity against Gram-positive bacteria than against Gram-negative bacteria and fungi. A correlation between cation lipophilicity and antimicrobial activity was detected, and only in some cases was their efficacy against Gram-positive strains comparable to that of BE chloride. Additionally, despite incorporating a relatively non-toxic anion, the mandelate-based ILs displayed higher cytotoxicity toward mammalian cells than their corresponding chloride analogues.

Considering the biocompatibility and biological activity of mandelic acid, J. Pernak et al. subsequently investigated a novel series of ILs derived from this α-hydroxy acid in combination with various piperidinium cations as antimicrobial agents against bacteria and fungi [113]. Piperidinium is a biocompatible and biodegradable cation present in other antimicrobial ILs with counterions of natural origin, such as theophyllinate anion [114].

**Scheme 4:**
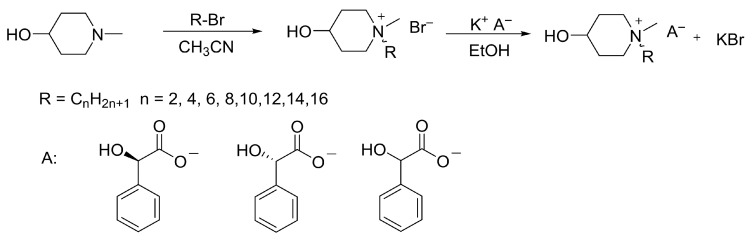
Synthesis of piperidinium madelate ILs.

The authors reported three homologous series of amphiphilic ILs derived from (S)-mandelate, (R)-mandelate and (RS)-mandelate, combined with 1-alkyl-4-hydroxy-1-methyl-piperidinium cations bearing alkyl chains ranging from C2 to C16 (Scheme 4). Structure-property-activity relationships were systematically evaluated to propose novel ILs with optimized pharmacodynamic and pharmacokinetic profiles.

The synthesis of these ILs was successfully performed in two steps (Scheme 4). First, 4-hydroxy-1-methylpiperidine was quaternized with different bromoalkanes, yielding mixtures of geometric isomers that could not be separated. This was followed by an anion-exchange process with potassium mandelate (S, R, and racemic forms).

All these salts, regardless of the anion chirality, were ILs and some of them (C_4_ to C_10_) RTILs. The physicochemical characterization of specific rotation, density, refractive index, surface activity, including adsorption efficiency, and solubility in different solvents, indicated that these properties were predominantly influenced by the alkyl chain length rather than by anion chirality.

A similar trend was observed for antimicrobial properties, with alkyl chain length emerging as the primary determinant of activity. Only ILs bearing alkyl chains of 10 to 16 carbon atoms displayed antibacterial and antifungal activity, with the C_14_ and C_16_ derivatives showing the highest potency. Consistent with other ammonium-based ILs and conventional quaternary ammonium compounds, the C_14_ and C_16_ analogs were more active against Gram-positive bacteria than against Gram-negative bacteria and fungi [69]. Overall, their activity against Gram-positive strains was only comparable to that of quaternary halides. However, as highlighted by the authors, the observed correlation between surface properties, amphiphilicity and antimicrobial effect may provide valuable guidance for the rational design of more potent antimicrobials.

**Figure 14:**
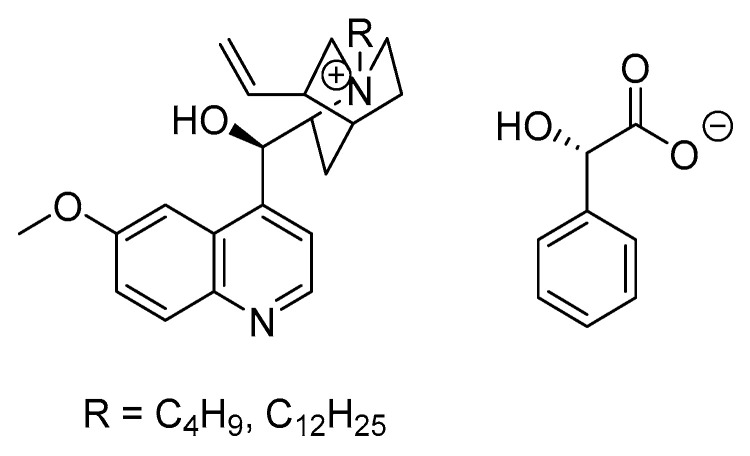
Quinine-based ILs with *S*-mandelate anion.

S-Mandelic acid was also selected by the same authors as an anionic precursor for the incorporation of N-alkyl quinine cations into ILs [115]. Given the well-documented antimalarial, antiviral, and even antifeedant properties of the quinine free base, these ILs (Figure 14) can be considered as multifunctional compounds from a biological perspective. However, the biological study conducted by the authors focused exclusively on antifeedant activity, which was found to be significantly higher than that of the quinine-free base.

**Figure 15:**
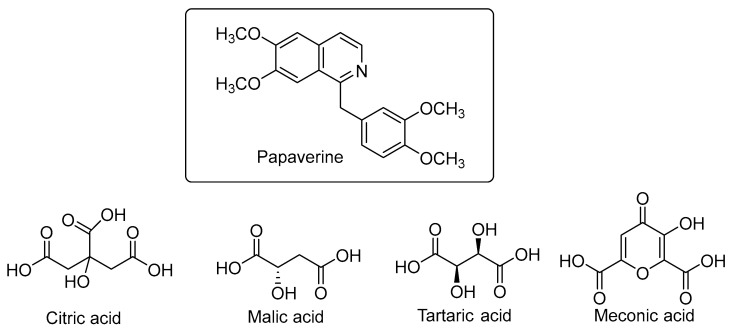
Papaverine and hidroxycarboxylic components of protic ILs.

To improve the pharmacokinetic properties of papaverine, an alkaloid with antispasmodic activity, it was combined with several naturally occurring hydroxycarboxylic acids, including citric, malic, tartaric, and meconic acids (Figure 15), to provide a small series of protic ILs. Most of these salts were obtained as amorphous solids with T_g_ values below 51 °C [116]. Potentiometric, photometric, and NMR aggregation studies conducted on papaverine citrate demonstrated enhanced dissolution rate, apparent aqueous solubility, and suspension stability compared to papaverine hydrochloride, the commercially available form of the API.

**Scheme 5:**
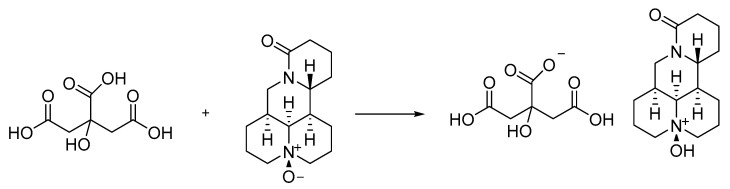
Synthesis of oxymatrinium citrate.

Citric acid was also recently combined with oxymatrine, a naturally occurring N-oxide analogous to matrine that exhibits a broad pharmacological profile, yielding oximatrinium citrate ([OMT][CA]), a novel IL with antibacterial properties and potential applications in food industry, biomedicine and materials science, along with a low environmental impact [117]. The [OMT][CA] IL was synthesized in an aqueous medium by mixing equimolar amounts of oxymatrine and citric acid (Scheme 5) and was isolated in its pure form with low water content (3.2%) through the careful removal of the solvent under vacuum, affording a RTIL.

The spectroscopic study (FT-IR and NMR spectroscopy) carried out by the authors confirmed the transfer of a proton from the carboxyl group of citric acid to the oxygen atom of the N-oxide in oxymatrine, enabling the establishment of an ionic interaction between both species.

Thermodynamic and kinetic studies, together with molecular dynamics and quantum calculations revealed that the [OMT][CA] IL displays a single Tg of −23.4 °C and strong intermolecular interactions, demonstrating non-Newtonian flow characteristics.

**Figure 16:**
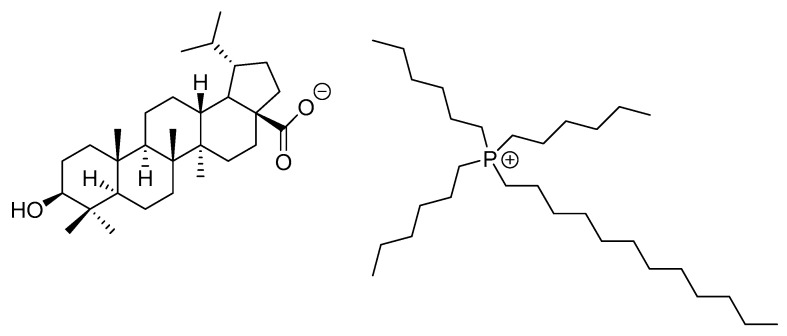
Trihexyltetradecylphosphosphonium betulinate.

Betulinic acid is a naturally occurring cyclic triterpenoid with a variety of chemotherapeutic properties, covering antiviral, antibacterial, antiparasitic and anticancer activities [118]. However, its high lipophilicity and low water solubility limit its clinical application. The formation of salts by combining the betulinate anion with different organic cations significantly improved both the water solubility and biological activity of betulinic acid [119,120,121]. Most of these salts contain ammonium cations and are solid at room temperature, showing relatively high melting points. In contrast, only tetradecyltrihe-xylphosphosphonium betulinate ([P_6,6,6,14_] [Bet]) has been reported as a RTIL (Figure 16). This betulinic-based IL, designed as a potential synergistic antitumor agent [121], demonstrated moderate antiproliferative activity in vitro and is notable for its selectivity against certain tumor cell lines, as well as its reduced toxicity to healthy cells compared with both ammonium salts and the parent betulinic acid.

**Figure 17:**
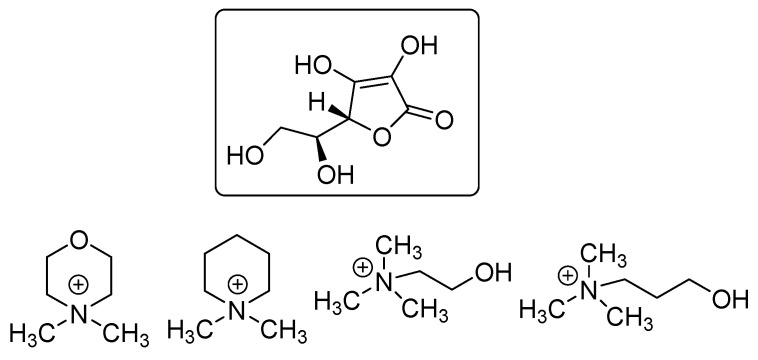
Structure of L-ascorbic acid and cations included in antioxidant RTILs.

The L-ascorbic acid, commonly known as vitamin C, is a water-soluble polyhydroxylated γ-lactone with antioxidant properties. Using this natural antioxidant, K. Czerniak synthesized a series of thermally stable RTILs via neutralization with the corresponding quaternary ammonium hydroxides (Figure 17). These ILs retain the antioxidant properties of the precursor acid, with their free radical scavenging efficacy modulated by the nature of the cation [122].

#### 2.3.2. Physicochemical Properties of Bioactive Hydroxy Acid-Based ILs

J. Pernak et al. [111] studied several ILs composed of long-alkyl-chain quaternary ammonium cations, including DDA, BE (a C_12_/C_14_ mixture in a 60%:40% ratio), and DOM, combined with the three available mandelate anions (S-Man, R-Man, and the racemic anion, Man). These salts (Figure 13) were described as waxes or RTILs, with macroscopic physical behavior primarily governed by the identity of the cation, while rheology, thermal stability, and interfacial packing were modulated by the anion. All of them are stable to air and moisture and miscible with water and organic solvents of varying polarity.

All mandelates in the DDA series exhibit low densities (ρ = 0.952 g·mL^−1^) and moderate viscosities, ranging from 3.68·10^3^ cP for [DDA][R-Man] to 5.73·10^3^ cP for [DDA][Man]. In contrast, mandelates in the BE series are denser (ρ = 1.015 g·mL^−1^) and significantly more viscous, reaching 18.40·10^3^ cP for the racemic form ([BE][Man]) and 22.40·10^3^ cP for the S-enantiomeric form ([BE][S-Man]), clearly reflecting the larger size and higher polarizability of the benzalkonium cation. Additionally, the three mandelates of DOM are described as waxes at room temperature, although density and viscosity data were not reported.

The calorimetric analysis reveals T_g_ values ranging from −22.1 °C to −51.0 °C. Notably, in the case of DDA and BE mandelates, the pure enantiomers clearly exhibit higher T_g_ values than the corresponding racemic mixtures. The authors attribute this behavior to more efficient and faster molecular packing within the system.

Thermal stability varies on both the cation and the anion chirality. ILs containing the DOM cation are the most thermally robust, with T_d_ values ranging from 266 °C for [DOM][R-Man] to 286 °C for [DOM][Man], whereas ILs in the BE series are the least stable, with T_d_ values ranging from 207 °C for [BE][Man] to 238 °C for [BE][S-Man]. Overall, DOM cation confers the greatest thermal robustness, followed by the DDA cation, while the BE cation exhibits narrower degradation intervals and includes the least thermally stable system.

The authors also studied the surface activity of these nine ILs. All of them reduced the water surface tension (72.8 mN·m^−1^) at the CMC. The CMC data revealed that the cation plays a determining role in interfacial behavior, with ILs in the BE and DOM series displaying the highest values, around 36 mN·m^−1^, whereas ILs of DDA series exhibited the lowest values, around 26 mN·m^−1^.

In contrast, no significant differences were detected between enantiomers and racemic forms. Overall, all compounds behave as surfactants; however, ILs in the DDA series, characterized by stronger hydrophobic cationic interactions, are more effective at reducing surface tension.

The same research group [113] characterized three homologous series of ILs derived from the three mandelate forms mentioned above (S-Man, R-Man and Man) and several 1-alkyl-4-hydroxy-1-methylpiperidinium cations, in which the alkyl substituent varies from an ethyl to a hexadecyl group (Scheme 4). The authors found that some of the salts bearing an ethyl chain are solids at 25 °C, with T_m_ values below 86 °C, whereas all derivatives with C_4_–C_10_ chains are RTILs, and those containing C_12_–C_16_ chains are highly viscous greases.

DSC analysis shows T_g_ values ranging from −5 °C for [C_16_H_33_][Man] to −17 °C for [C_14_H_29_][R-Man], confirming the amorphous nature of most compounds. The specific rotation, [α]D^20^, reflects the chirality of the anion. For the (S)-mandelate series, values range from +57.219° in [C_2_H_5_][S-Man] to +32.241° in [C_16_H_33_][S-Man], whereas, for the (R)-mandelate series they range from −64.981° in [C_2_H_5_][R-MAN] to −29.548° in [C_16_H_33_][R-Man]. In contrast, (RS)-mandelate salts exhibit values close to zero, confirming their racemic composition. The decrease in optical rotation with increasing alkyl chain length follows a logarithmic trend.

The density at 20 °C decreases with increasing chain length, ranging from 1.1920 g·cm^−3^ for [C_4_H_9_][S-Man] to 1.0990 g·cm^−3^ for [C_10_H_21_][S-Man]. These values are higher than those reported for ILs with DDA and BE cations, an effect attributed to the presence of the hydroxyl group in the piperidinium cation. The refractive index at 20 °C ranges from 1.5327 for [C_4_H_9_][S-Man] to 1.5190 for [C_10_H_21_][S-Man], with minimal differences between enantiomers. This parameter may serve as an auxiliary indicator of chirality.

All these ILs are highly soluble in water and very polar organic solvents, and insoluble in nonpolar organic solvents. This behavior is associated with hydrogen-bonding capability provided by hydroxyl groups in both cation and anion. In addition, the studied ILs reduce the surface tension of water more effectively than their bromide analogous, displaying CMC values ranging from 28.3 mN·m^−1^ to 29.7 mN·m^−1^. Furthermore, the CMC decreases with increasing alkyl chain length, from 83.2 mmol·L^−1^ for [C_6_H_13_][S-MAN] to 2.63 mmol·L^−1^ for [C_16_H_33_][S-Man]. The authors conclude that the length of the cation alkyl chain modulates the surface properties of these ILs, which in turn determines their disinfectant activity.

The S-Man anion was also combined with two N-alkylpiperidinium cations derived from quinine, in which the N-alkyl fragment is a *n*-butyl or a *n*-dodecyl group, affording novel naturally derived ILs (Figure 14). Both salts, which were studied by J. Pernak et al. from a physicochemical perspective [115], are classified as RTILs.

DSC analysis revealed that the N-butyl analogue exhibits a T_m_ of 6 °C, whereas the N-dodecyl derivative shows a Tg value of 45 °C, significantly higher than those reported for other N-alkylpiperidinium analogues described by the same authors [113]. This result indicates reduced segmental mobility and enhanced structural rigidity.

Both RTILs display remarkable thermal stability, with T_d_ values of 279 °C for the N-butyl analogue and 344 °C for the N-dodecyl derivative. Regarding solubility, only the N-butyl analogue is water-soluble and compatible with aqueous formulations; however, both compounds are soluble in ethanol and insoluble in nonpolar organic solvents.

U. Holzgrabe et al. [116] demonstrated that the formation of protic ILs (PILs) by combining the alkaloid papaverine with natural hydroxycarboxylic acids, such as citric, malic, tartaric and meconic acids (Figure 16), modifies its physicochemical properties, thereby improving key parameters for pharmaceutical performance.

Three of the four PILs obtained (1:1 molar ratio cation:anion) are amorphous compounds and exhibit T_g_ ranging from 30 °C to 50 °C; however, papaverine meconate is a crystalline solid with a Tm of 88 °C. In contrast, papaverine itself and papaverine hydrochloride, the commercially available salt of the API, show melting points of 149 °C and 220 °C, respectively, highlighting the significant structural differences.

The loss of crystalline order, attributed to intramolecular hydrogen bonding involving α-hydroxycarboxylic acids, reduces lattice energy and enhances molecular mobility, which also explains the improved solubility. Quantitative data on dissolution rate and aqueous solubility were reported for papaverine citrate and papaverine malate. Both parameters are higher for these amorphous solids than for the crystalline solids, namely papaverine and papaverine hydrochloride.

Colloidal behavior was assessed for papaverine citrate using Dynamic Light Scattering (DLS) and zeta potential measurements, revealing the formation of nanoscale aggregates with a low polydispersity index (PDI ≈ 0.20 at 80 mM), compared with papaverine hydrochloride (PDI = 0.56 at 20 mM). In addition, marked electrostatic stabilization of colloidal system was observed, with zeta potential values of −22 mV for papaverine citrate versus +18 mV for the corresponding hydrochloride.

The viscosity of the suspension slightly increased for papaverine citrate (0.94 mPa·s) relative to papaverine hydrochloride (0.93 mPa·s), reflecting the influence of its greater aggregation capacity and steric complexity of the counterion.

A similar behavior is observed in oxymatrinium citrate ([OMT][CA]), another citrate-based protic IL, whose physicochemical profile mirrors many of the trends described for papaverine citrate. A recent study dedicated to the OMT-CA IL [117], shows that this IL remains amorphous over an exceptionally broad thermal interval, from −80 °C to 100 °C, with no evidence of crystallization. This wide crystallization free range confirms that the ionic structure of OMT-CA is highly disordered and sustained by electrostatic and hydrogen bonding interactions that readily reorganize. Thermogravimetric analysis indicates moderate stability, with a slight initial mass loss associated with residual moisture (≈3%) and no significant degradation up to approximately 120 °C, a behavior characteristic of hydroxycarboxylic acid-derived PILs. Moreover, the study shows that OMT-CA exhibits non-Newtonian rheological behavior, with viscosity decreasing as shear rate increases, indicating that the ionic interaction network reorganizes easily under mechanical stress. Interaction energy analysis supports this interpretation, showing that the ionic dimer displays a stronger interaction than the corresponding molecular dimer, in line with the predominance of stable electrostatic interactions within the IL.

The ascorbate-based RTILs synthesized by K. Czerniak [122] further demonstrated that the cation structure modulates the physicochemical properties of the system. Thus, combining the ascorbate anion with different quaternary ammonium cations, such as N,N-dimethylmorpholinium, N,N-dimethylpiperidinium, cholinium and its homologous N-(3-hydroxypropyl)-N,N,N-trimethylamonium (Figure 17), generates significant differences in glass transition temperature, thermal stability, and to a lesser extent in solvent compatibility.

The T_g_ values range from −5.54 °C for the morpholinium analogue to 12.21 °C for the piperidinium derivative. The lower T_g_ observed for morpholinium-based systems is attributed to the presence of an oxygen atom within the heterocyclic ring, which enhances molecular flexibility and reduces rigidity at low temperatures. In the case of the cholinium derivative and its propyl analogue, increasing the alkyl chain length raises T_g_ from 0.42 °C to 7.64 °C due to the enhancement of intermolecular interactions.

All the studied RTILs exhibit high thermal stability, with T_d_ values ranging from 225 °C to 238 °C, the heterocyclic-cation-based RTILs being the most stable. All compounds are fully soluble in water, methanol, and DMSO, while remaining insoluble in nonpolar or weakly polar organic solvents, although slight differences in miscibility were detected. Beyond solubility and thermal behavior, the cation structure also affects optical and packing properties. At 20 °C, morpholinium and piperidinium RTILs exhibit the highest refractive indices (1.539 and 1.542, respectively) combined with the lowest densities (1.22–1.23 g·cm^−3^), which may indicate greater polarizability per unit mass. In contrast, the cholinium RTIL and its propyl derivative show refractive index values around 1.526 and higher densities (up to 1.265 g·cm^−3^), consistent with stronger hydrogen-bonding networks that increase packing efficiency without enhancing optical response.

## 3. Physicochemical Highlights of the Reviewed Carboxylic Acid-Based ILs

The thermal and physicochemical behavior of carboxylic acid-based ILs is governed by the structural nature of the anion, its hydrogen-bonding capacity, and the conformational mobility of the ion pair. These factors determine their melting and glass-transition temperatures, thermal stability, viscosity and solubilization performance, as summarized in Table 1. In choline-based FAILs, the pronounced flexibility of aliphatic anions results in low transition temperatures, reduced packing efficiency and high fluidity, they are predominantly amorphous systems. This is consistent with the homologous series of choline carboxylate ILs, which show systematic decreases in density and surface tension and increases in ionic conductivity with increasing temperature and anion length, highlighting the interplay between conformational freedom and hydrogen-bonding strength in non-protic systems.

In contrast, ILs derived from phenolic anions exhibit higher melting points and significantly higher thermal stability, reflecting the rigidity of aromatic rings and the presence of extended hydrogen-bonding networks. Hydroxyacid-based ILs display predominantly amorphous behavior. The abundance of hydroxyl groups in both ions disrupts crystal packing and favors the amorphous structures with notable solubilization capacity. The OMT-CA PIL is fully consistent with this pattern, displaying a single glass-transition temperature and a moderated thermal stability characteristic of proton-transfer systems.

These families follow a coherent structure-property relationship. Aliphatic anions, due to their high conformational flexibility, tend to yield ILs with low T_m_ and T_g_ and high fluidity. Aromatic anions, being more rigid, produce higher T_m_, T_onset_ values and improved thermal stability. Polyhydroxylated anions generate amorphous liquids with low T_g_ and strong solvation ability. These trends are consistent with the broader distinction between protic and non-protic ILs. The proton-transfer systems tend to display lower thermal stability and enhanced amorphicity, whereas non-protic ILs based on quaternary cations generally present higher T_onset_ values and more sharply defined phase transitions.

## 4. Conclusions

The evidence gathered demonstrates that transforming APIs, particularly those derived from natural compounds, into ILs offers substantial advantages over their covalent or crystalline salt counterparts. These benefits include the elimination of polymorphism, significant improvements in solubility and membrane permeability, two key determinants of bioavailability, and the potential to create therapeutic synergies through the combination of complementary bioactive species.

The fine modulating cation-anion combinations enables precise tuning of properties such as hydrophobicity, hydrogen bonding capacity, membrane permeability, and micelle formation, thereby enhancing the biological and pharmacokinetic profiles of precursor molecules across a broad range of bioactivities, including antimicrobial, antifungal, antiparasitic, anticancer, antioxidant, anti-inflammatory applications, as well as drug delivery field.

A critical analysis of toxicity and biocompatibility further highlights the decisive role of both anion and cation structures in determining cytotoxicity, ecotoxicity, and safety profiles. While certain ILs derived from fatty acids or cholinium display low toxicity and favorable biodegradability, others require thorough assessment due to increased persistence or adverse effects in cellular and aquatic models.

Notably, numerous bioinspired ILs based on carboxylic acids, including fatty, phenolic and hydroxy acids and incorporating API-derived cations such as choline, agmatine, matrine, oxymatrine, chloroquine, primaquine, papaverine, as well as other alkylammonium or alkylphophonium cations, generally exhibit enhanced solubility, stability, and therapeutic potential compared to their parent compounds, ultimately improving drug efficacy.

Considering the growing demand for more effective and innovative therapeutic strategies, the development of carboxylate-based ILs is expected to remain a highly attractive area of research in the coming years.

## Figures and Tables

**Table 1:** Comparative summary of the carboxylic acid-based ILs described in this review.

Type of IL	Structural Characteristics	Physicochemical Properties
FAILs	Aliphatic anions with high conformational mobility and reduced packing efficiency. Cations include choline or simple ammonium species.	Low melting points (26–80 °C). Very low T_g_ (down to −74 °C). Thermal stability T_d_: 166–210 °C. Moderate solubility and amphiphilic behavior. Viscosity dependent on alkyl-chain length. Additional thermophysical properties (ρ, γ, σ, V^M^) vary systematically with anion size.
Phenolic acid-based ILs	Aromatic anions capable of extensive hydrogen bonding.	Broad thermal behavior: from RTILs to solids with higher melting points. Tg (13–19 °C). Thermal stability T_d_: 150–308 °C. High water solubility (especially cholinium salts).
Hydroxy acid-based ILs	Polyfunctional anions with multiple OH groups and, in some cases, chirality or chelating capability.	T_g_: −51–50 °C. T_m_: 40–90 °C. T_d_: 120–286 °C. High solubility, predominantly amorphous.
PILs vs. non-PILs	PILs: Cations derived from amines or alkaloids.	PILs: Lower thermal stability and enhanced amorphicity due to extensive H-bonding.
	Non-PILs: Quaternary ammonium or phosphonium cations.	Non-PILs: Higher thermal stability, defined phase transitions and rigid ion-pair organization.

## Data Availability

No new data were created or analyzed in this study. Data sharing is not applicable to this article.

## Abbreviations

The following abbreviations are used in the manuscript.

APIs	active pharmaceutical ingredients
FAILs	fatty acid-based ionic liquids
API-ILs	active pharmaceutical ingredient ionic liquids
HAs	hydroxy acids
CMC	critical micelle concentration
ILs	ionic liquids
DES	deep eutectic solvent
MBC	minimal bactericidal concentration
DILs	dicationic ionic liquids
PILs	protic ionic liquids
DSC	differential scanning calorimetry
RTILs	room-temperature ionic liquids
EC50	half maximal effective concentration
